# BV/ODV-E26 is a conserved baculoviral inhibitory factor for optimizing viral virulence in lepidopteran hosts

**DOI:** 10.1016/j.isci.2024.111723

**Published:** 2024-12-31

**Authors:** Hiroyuki Hikida, Ryuhei Kokusho, Susumu Katsuma

**Affiliations:** 1 Institute for Chemical Research, Kyoto University, Gokasho, Uji, Kyoto 611-0011, Japan; 2 Department of Agricultural and Environmental Biology, Graduate School of Agricultural and Life Sciences, The University of Tokyo, 1-1-1, Yayoi, Bunkyo-ku, Tokyo 113-8657, Japan

**Keywords:** Transcriptomics, Virology

## Abstract

Alphabaculoviruses induce abnormal behavior in lepidopteran larval hosts. A baculoviral gene, *bv/odv-e26*, is crucial for behavioral manipulation in *Bombyx mori* larvae by Bombyx mori nucleopolyhedrovirus (BmNPV). However, how *bv/odv-e26* fulfills its role in this phenotype remains largely unknown. In this study, we found that the overexpression of BmNPV *bv/odv-e26* delayed viral infection in cultured cells and decreased pathogenicity in *B. mori* larvae. We also discovered that homologs of *bv/odv-e26* are conserved more widely in alphabaculoviruses than previously thought. The inhibitory activity was demonstrated in *bv/odv-e26* homologs of phylogenetically close and distant baculoviruses, indicating conserved inhibitory function among alphabaculoviruses. Furthermore, locomotory analyses revealed that *bv/odv-e26* increased larval locomotory activity but had little effect on the timing of abnormal behavior initiation. Collectively, our findings demonstrate that *bv/odv-e26* is a baculoviral inhibitory factor that is widely conserved in the genus *alphabaculovirus* and may reduce viral virulence for successful host behavioral manipulation.

## Highlights

• BV/ODV-E26 inhibits viral pathogenicity to *Bombyx mori* larvae
• This protein is conserved in most alphabaculoviruses with its suppressive function
• This protein prolongs host abnormal behavior but has little effect on its onset

## Graphical abstract

**Figure:**
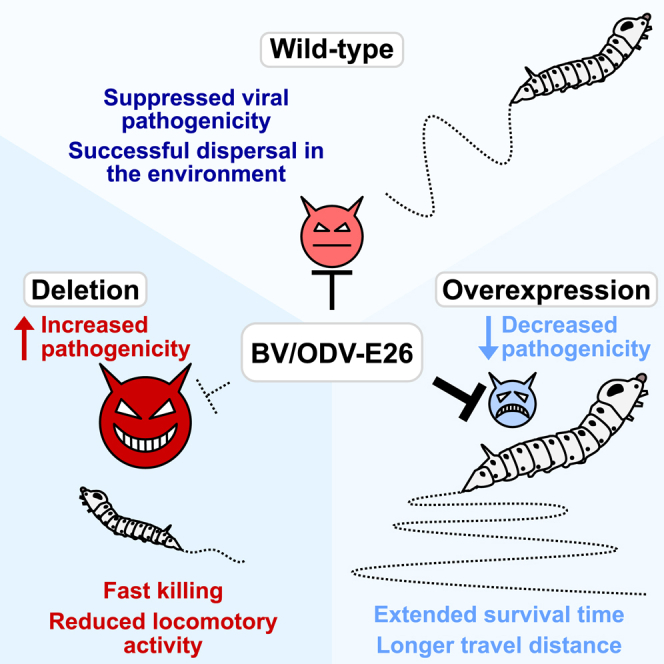

## Introduction

The family *Baculoviridae* is a group of insect-specific large double-stranded DNA viruses. Their genomic DNAs are packaged in rod-shaped, enveloped virions and further included in occlusion bodies (OBs).^1^ Based on the morphology of OBs and their host insect species, the family is phylogenetically divided into four genera: *Alphabaculovirus*, *Betabaculovirus*, *Gammabaculovirus*, and *Deltabaculovirus*.^2^^,^^3^^,^^4^ Among them, *Alphabaculovirus*, also known as lepidopteran nucleopolyhedroviruses (NPVs), has been extensively investigated and is further subdivided into groups I and II.^5^ Alphabaculoviruses are unique among viruses to produce two types of virions for systemic infection: occlusion-derived viruses (ODVs) and budded viruses (BVs).^6^ Orally inoculated OBs dissolve in an alkaline environment of the host midgut and release ODVs to invade the host. BVs subsequently spread viral infection to various tissues through tracheal system and hemolymph of insects.^7^^,^^8^^,^^9^ At a late stage of infection, the infected cells produce a large number of OBs and the larval body is degraded by the virus-encoded cathepsin and chitinase.^10^^,^^11^ Finally, progeny virus-containing OBs are spread into the environment.

Viral gene expression is tightly regulated in alphabaculovirus-infected cells. After viral entry, immediate-early genes are expressed, and their products initiate virus infection. Delayed-early genes are expressed with the support of some immediate-early genes and their products facilitate viral genome replication and expression of late genes.^12^ Late genes are transcribed by a viral RNA polymerase and encode proteins that comprise the nucleocapsid.^13^^,^^14^^,^^15^ Some preceding genes support the expression of very late genes, *polh* and *p10*,^16^ which encode extraordinarily highly expressed proteins that comprises OBs and facilitates OB release into the extracellular environment, respectively.^1^^,^^17^

A baculoviral gene, *bv/odv-e26*, encodes a protein with limited homology to other known proteins. This protein does not possess notable domains except for the coiled-coil domain. *bv/odv-e26* homologs were identified only in the genomes of group I alphabaculoviruses.^5^^,^^18^ Originally, this gene was found in Autographa californica multiple nucleopolyhedrovirus (AcMNPV), a prototype of alphabaculoviruses, and is implicated in late gene expression.^13^ The encoded protein called Ac16, interacts with a viral *trans*-activator IE1 presumably through the coiled-coil domain.^19^ Bombyx mori nucleopolyhedrovirus (BmNPV), a close relative of AcMNPV, encodes a homologous protein called Bm8, which colocalizes with IE1 and genomic enhancer region *homologous region*s (*hr*s).^20^^,^^21^ This complex exhibits punctate localization in the nucleus, suggesting that Bm8 is also involved in the regulation of gene expression. Biochemical analysis also suggested that Ac16 or Bm8 interacts with another viral protein (i.e., FP25K) and some host proteins, respectively, but the biological roles of these interactions were currently unknown.^22^^,^^23^

In *Bombyx mori* larvae, a mutant BmNPV with *bv/odv-e26* deletion enhances viral replication in multiple tissues.^24^^,^^25^ The deletion of *bv/odv-e26* also results in a fast-killing phenotype and less enhanced locomotory activity of the infected *B. mori* larvae.^25^ These results suggest that *bv/odv-e26* is a crucial factor for systemic infection and subsequent host behavioral manipulation of group I alphabaculoviruses. However, how *bv/odv-e26* regulates viral infection in infected larvae remains largely unknown. In the present study, by combining the pathogenic properties of *Bm8* mutants in *B. mori* larvae and cultured cells, we found that BV/ODV-E26 reduce viral virulence in larval hosts. In addition, we identified orthologous genes of *bv/odv-e26* from group II alphabaculoviruses. Mutagenesis experiments revealed the functional importance of two conserved residues in the coiled-coil domain of the Bm8 protein, which was observed in an orthologous protein from a phylogenetically distant alphabaculovirus. We further investigated the locomotory pattern and activity of *B. mori* larvae infected with *Bm8* mutant viruses. This gene was dispensable for the induction of abnormal host behavior but required to enhance host locomotory activities with an extended period of abnormal behavior. Taken together, these findings indicate that *bv/odv-e26* orthologs are commonly utilized to reduce viral virulence in host insects, which may contribute to the successful dispersal of alphabaculoviruses in the environment.

## Results

### *Bm8* overexpression delays viral infection in both *B. mori* larvae and cultured cells

**Figure 1:**
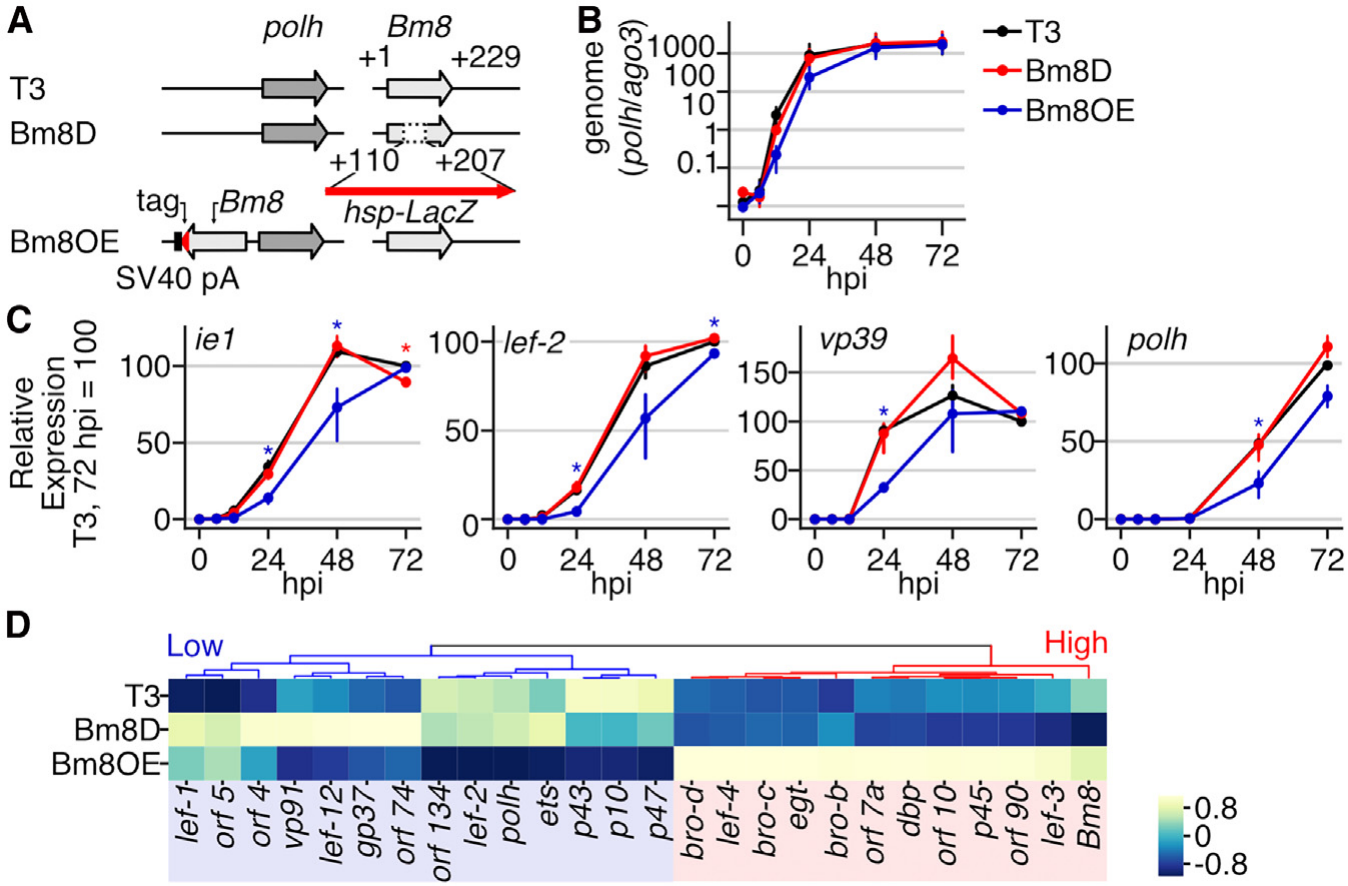
Phenotypes of a *Bm8*-overexpressing BmNPV in *B. mori* cultured cells (A) A Schematic image of recombinant viruses. In Bm8OE, a DYKDDDDK tag was attached to the C-terminus of the second copy of the *Bm8* product. (B) Viral genome replication in infected cells (*n* = 3). (C) Temporal expression of *ie1*, *lef-2*, *vp39*, and *polh*, representing immediate-early delayed-early, late, and very-late genes, respectively. Blue and red asterisks indicate that Bm8OE and Bm8D show significant differences from the other two viruses, respectively. *p* < 0.05, Tukey’s honestly significant difference (HSD) test. (B and C) Data are represented as mean and 95% confidence intervals. (D) Clustered heatmap of viral gene expression at 48 h post infection (hpi). Genes showing significantly different expression with >2-fold change are shown (*n* = 2). Blue and red indicate clusters in which genes show low and high expression in Bm8OE-infected cells, respectively. The results of the third replicate are shown in Figure S1.

Previous studies revealed that *Bm8* deletion enhances viral virulence in *B. mori* larvae.^24^^,^^25^ In the present study, we generated a recombinant BmNPV with two copies of *Bm8* (*Bm8-*overexpressing virus, Bm8OE; Figure 1A), and investigated its phenotypes in *B. mori* cultured cells and larvae. Whereas the viral genome replication patterns were similar between *Bm8*-deleted (Bm8D) and wild-type (T3) viruses, the level of viral replication in Bm8OE-infected cells was slightly lower at an early stage of infection and became similar to the other two viruses at a late stage (Figure 1B). The expression levels of *ie1*, *lef-2*, *vp39*, and *polh*, representative genes from immediate-early, delayed-early, late, and very late genes, respectively, were significantly lower in Bm8OE-infected cells than those of the other two viruses (Figure 1C). The global expression pattern of viral genes was further investigated by RNA sequencing at 48 h post infection (hpi) (Figures 1D and S1). Viral genes with differential expression patterns were largely classified into two groups by expression levels in Bm8OE-infected cells. Although some representative genes of the viral infection were missing from the differentially expressed genes presumably due to the detection power, highly expressed genes in Bm8OE-infected cells included early genes (i.e., *bro* genes and *egt*), whereas those with low expression included very late genes (i.e., *polh* and *p10*). Collectively, these results indicated that *Bm8* overexpression delays the progression of viral infection in host cells.

**Figure 2:**
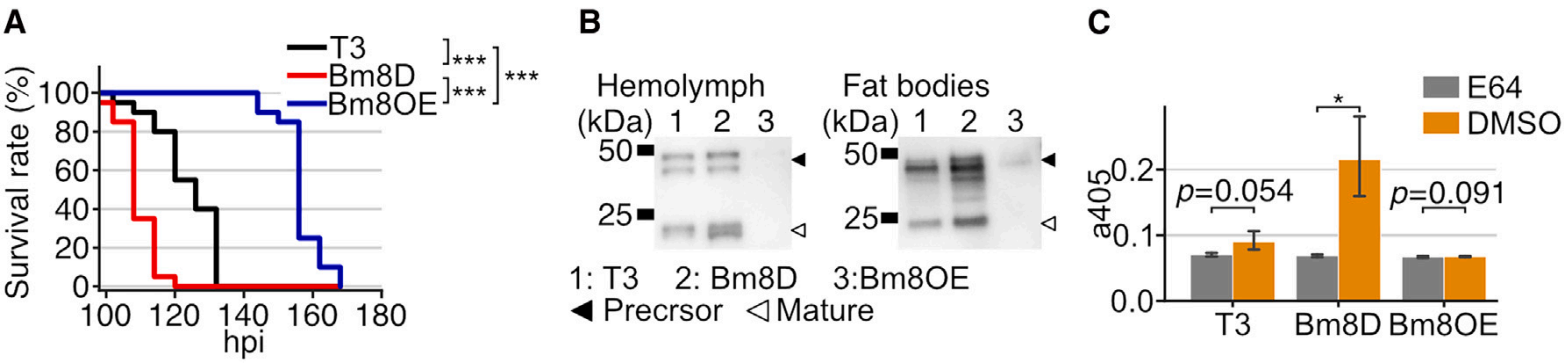
Phenotypes of a *Bm8*-overexpressing BmNPV in *B. mori* larvae (A) Survival curves of infected larvae (*n* = 20, ∗∗∗*p* < 0.005, pairwise log rank test). LT_50_ was 126, 108, and 156 h for T3-, Bm8D-, and Bm8OE-infected larvae, respectively. (B) V-CATH accumulation and expression in larval hemolymph and fat bodies, respectively, at 90 hpi. (C) V-CATH activity in the larval hemolymph (*n* = 3). E-64 was used as a cysteine protease inhibitor. Data are represented as mean with 95% confidence intervals. (∗*p* < 0.05, one-side paired-samples *t*-test).

We further investigated phenotypes of Bm8OE in *B. mori* larvae. Bm8OE significantly extended host survival time, whereas Bm8D showed a significant fast-killing phenotype compared with T3 (Figure 2A). All larvae infected with Bm8OE died, indicating that *Bm8* overexpression delays but does not completely inhibit the progression of viral infection. In some alphabaculoviruses, including BmNPV, viral cathepsin (V-CATH) is crucial for viral virulence and host survival time.^26^ Therefore, V-CATH accumulation was examined in the hemolymph and fat bodies of infected larvae. The expression and accumulation of V-CATH were lower in the fat bodies and hemolymph of Bm8OE-infected larvae than those infected with T3 and Bm8D. Mature V-CATH was not detected in Bm8OE-infected larvae at 90 hpi (Figure 2B). Whereas the hemolymph of T3-and Bm8D-infected larvae showed slight and high V-CATH activity, respectively, V-CATH activity was not detected in the hemolymph of Bm8OE-infected larvae (Figure 2C). These results demonstrated that *Bm8* overexpression results in delay of infection in host cells, which further prolongs viral infection in host larvae.

### Conserved amino acid residues in the coiled-coil domain are required for the suppressive function of the Bm8 protein

**Figure 3:**
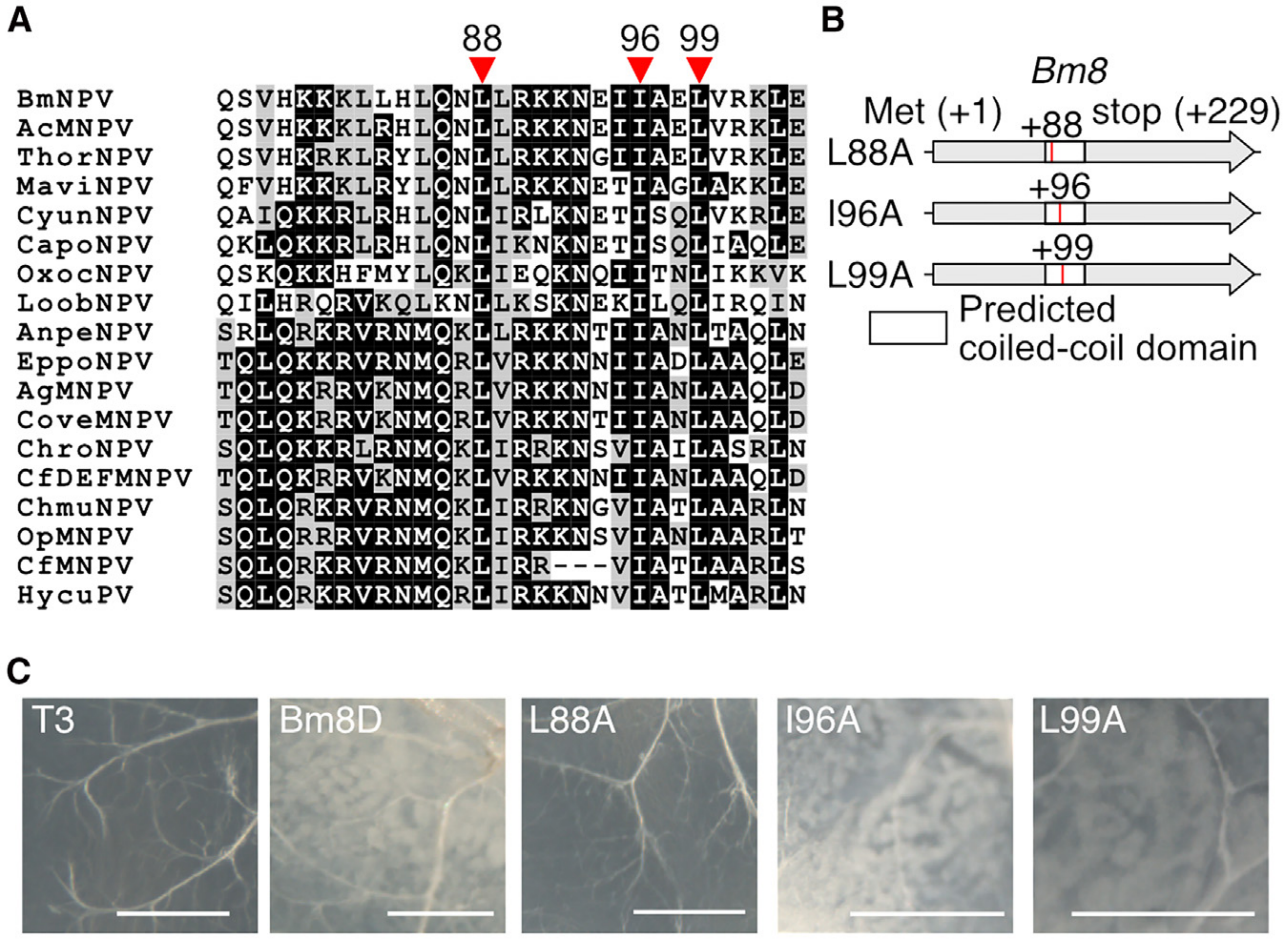
Identification of the conserved residues required for the Bm8 protein function (A) Alignment of the coiled-coil domains of Bm8 homologs found in group I alphabaculoviruses. Red arrowheads indicate residues conserved in all homologs. Residue numbers in the Bm8 protein are indicated above the arrowheads. The abbreviations for viruses are listed in Table S2. (B) A Schematic image of point mutations introduced in recombinant viruses. Red lines indicate the position of point mutations in the coiled-coil domain. (C) Microscopic images of infected MSGs at 90 hpi. White blobs are OB-containing cells. Scale bars: 200 μm.

To determine the functionally important region in the Bm8 protein, we searched functional domains of Bm8 and detected the coiled-coil domain only (Figure S2). This domain is required for interaction with IE1,^21^ suggesting its importance for Bm8 to suppress viral infection. We focused on this domain and identified three potential functional residues that were well conserved in Bm8 homologs found in group I alphabaculoviruses (Figure 3A). Three recombinant BmNPVs were generated, in which a point mutation was introduced at the conserved amino acid residues. (Figure 3B). These recombinant BmNPVs were inoculated to *B. mori* larvae, and OB formation was investigated in the middle silk glands (MSGs) (Figure 3C). As reported previously,^24^^,^^25^ few OBs were observed in the MSGs of T3-infected larvae, whereas many OBs were observed in the MSGs of Bm8D-infected larvae. The introduction of an amino acid substitution at the leucine 88 did not affect OB production in MSGs. In contrast, mutations in the isoleucine 96 or leucine 99 increased OB production, similar to that in the Bm8D phenotype. These results indicated that two residues, the isoleucine 96 and leucine 99 in the coiled-coil domain, are required for the suppressive function of the Bm8 protein in *B. mori* larvae.

**Figure 4:**
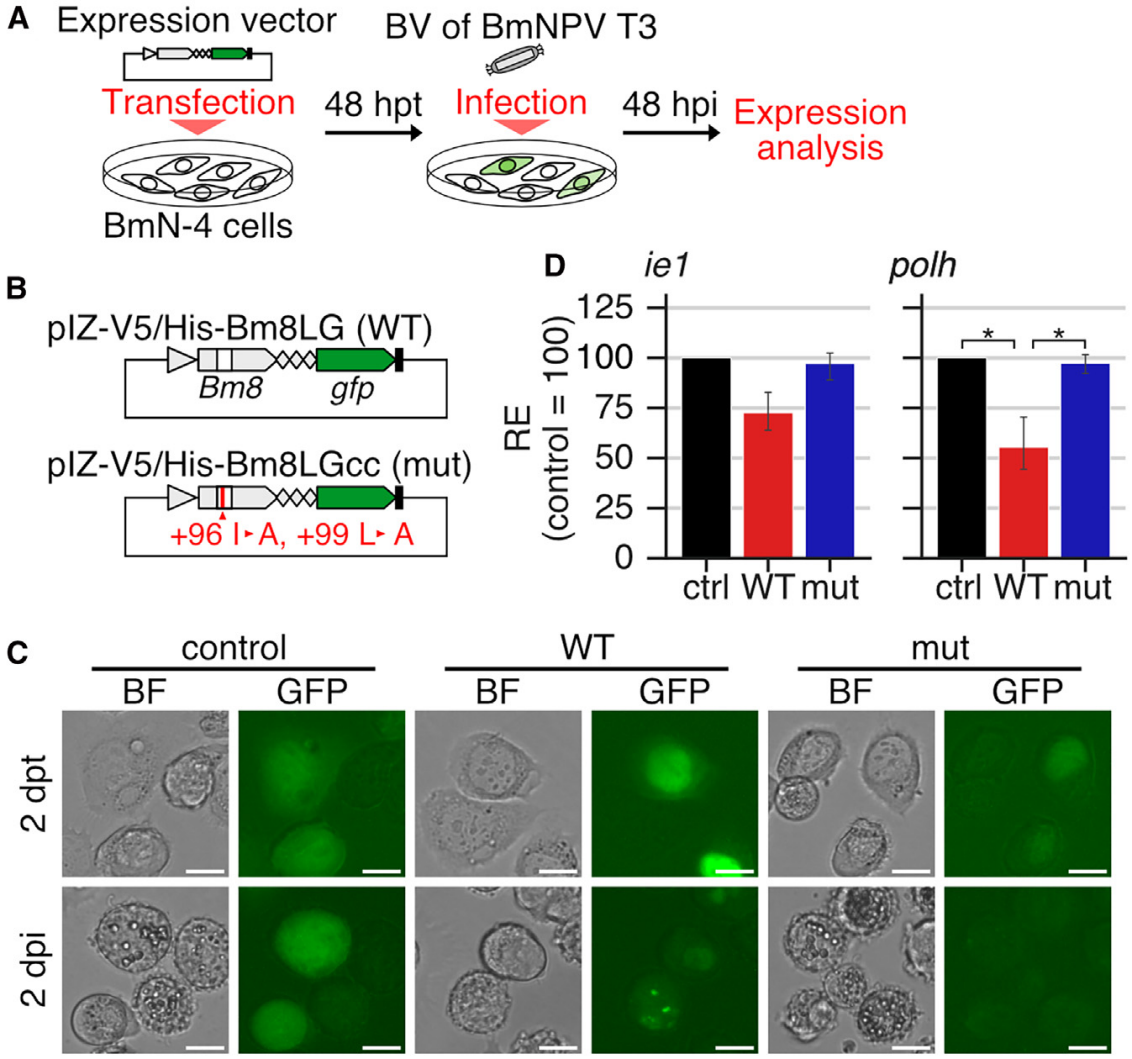
Contribution of the conserved residues of Bm8 in its suppressive function (A and B) Schematic images of (A) experimental procedure and (B) plasmid vectors used in the experiments. (B) Red line indicates point mutations in the coiled-coil domain. (C) GFP fluorescence pattern in control-transfected, wild-type-transfected, and mutant-transfected BmN-4 cells at 48 h post transfection (hpt), followed by T3 infection at 48 hpi. Scale bars: 20 μm. (D) Relative expression of *ie1* and *polh* in plasmid-transfected cells at 48 hpi. The expression level in cells transfected with the control vector (pIZ-GFP) is set as 100. Data are represented as mean with 95% confidence intervals (*n* = 3). (∗*q* < 0.05, paired-samples *t*-test followed by Benjamini-Hochberg correction).

The importance of isoleucine 96 and leucine 99 in the Bm8 protein function was further investigated using *B. mori* cultured cells. Plasmid vectors expressing green fluorescent protein (GFP)-fused wild-type Bm8 or GFP-fused Bm8 derivatives with the alanine 96 and alanine 99 were constructed and transfected into BmN-4 cells, followed by T3 infection (Figures 4A and 4B). GFP fluorescence in cells transfected with the wild-type vector was observed uniformly in the nucleus without virus infection and changed to punctate loci in the nucleus after infection (Figure 4C). In contrast, transfection of the mutant vector resulted in weak and almost no fluorescence before and after infection, respectively. The expression level of *polh* was significantly lower in cells transfected with the wild-type vector than control cells. This decrease was not observed in mutant vector-transfected cells (Figure 4D). Similar trends were obtained in *ie1* expression, although the differences were not significant (Figure 4D). These results showed that isoleucine 96 and leucine 99 are required for the suppressive function of the Bm8 protein in *B. mori* cultured cells.

### *bv/odv-e26* and its suppressive function are widely conserved in alphabaculoviruses

**Figure 5:**
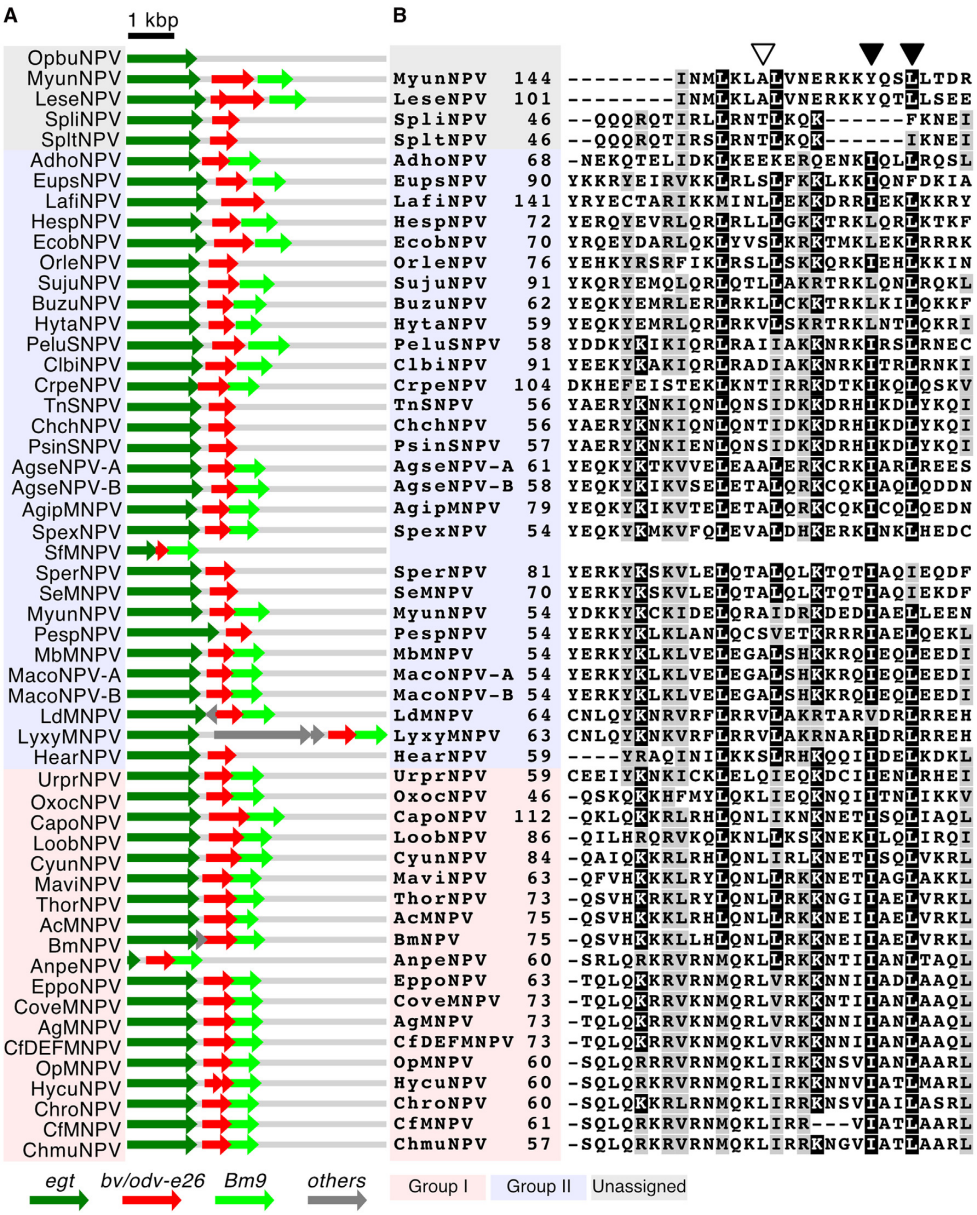
Identification and characterization of *bv/odv-e26* homologs (A) Distribution of *bv/odv-e26* homologs in alphabaculovirus genomes. (B) Alignment of coiled-coil domains. White arrowhead indicates residue 88 of Bm8. Black arrowheads indicate residues 96 and 99. (A and B) Red, blue, and gray background colors indicate group I and II and unassigned alphabaculoviruses, respectively.

Our results suggest that *Bm8* has an inhibitory role in viral propagation in infected cells, which thereby optimizes viral systemic infection in host larvae. Homologs of *bv/odv-e26* are considered to be conserved only in group I alphabaculoviruses, although group II alphabaculoviruses also exhibit systemic infection to manipulate host behavior.^18^^,^^27^ A previous study indicated that some group II alphabaculoviruses have genes located between *egt* and *Bm9* homologs, whose product sizes are similar to that of *bv/odv-e26*.^28^ Because many alphabaculoviruses harbor *egt* and a large number encode *Bm9* homologs, we hypothesize that phylogenetically distant *bv/odv-e26* homologs are located between the two genes in some genomes of group II alphabaculoviruses. To examine this hypothesis, all open reading frames (ORFs) between *egt* and *Bm9* homologs were selected, and alphabaculovirus genomes were searched for their homologous proteins (Figure S3). Most alphabaculoviruses harbored a homolog of *bv/odv-e26* between *egt* and *Bm9* (Figure 5A). Some alphabaculoviruses did not possess *Bm9* homologs but harbored *bv/odv-e26* homologs downstream of *egt*. Among the 54 alphabaculovirus genomes examined, a *bv/odv-e26* homolog was not detected only in the genome of Operophtera brumata nucleopolyhedrovirus (OpbuNPV), which is one of the most ancient types of alphabaculoviruses.^29^

Deduced amino acid sequences of BV/ODV-E26 homologs were aligned, and their phylogenetic tree was constructed. The tree topology was largely consistent with that constructed using 38 baculovirus core genes, and homologs in group I and II alphabaculoviruses were phylogenetically separated (Figure S4). The alignment of deduced amino acid sequences of these homologs showed highly conserved coiled-coil domains (Figure 5B). Particularly, amino acid residues corresponding to isoleucine 96 and leucine 99 in the Bm8 protein, essential for its suppressive function, were conserved in most group I and II alphabaculoviruses. In some group II alphabaculoviruses, the corresponding residues were replaced with structurally similar amino acids (i.e., valine, leucine, or isoleucine). Leucine 88, dispensable for Bm8 function (Figure 3C), was not conserved in group II alphabaculoviruses. These results strongly suggest that *bv/odv-e26* homologs have similar suppressive functions.

**Figure 6:**
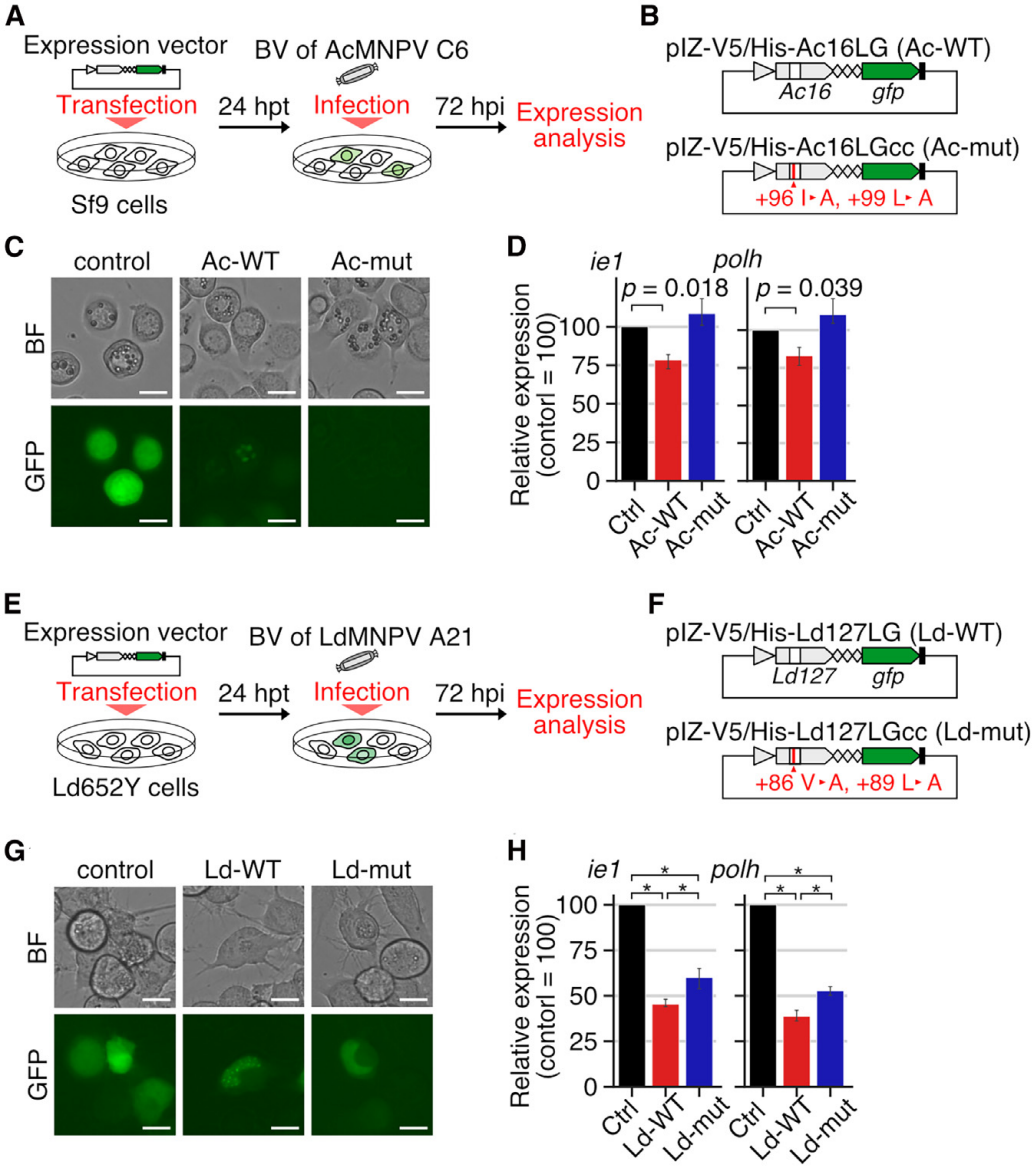
Functional analysis of *bv/odv-e26* homologs in susceptible cell lines (A–D) Schematic images of (A) experimental procedure and (B) plasmid vectors for (C) and (D). (C) AcMNPV-infected Sf9 cells at 72 hpi. Scale bars: 20 μm. (D) Relative expression of *ie1* and *polh* in plasmid-transfected Sf9 cells after AcMNPV infection. *p* values were calculated by paired-samples *t*-test and corrected by using the Benjamini-Hochberg method but significant differences were not detected after correction. (E–H) Schematic images of (E) experimental procedure and (F) plasmid vectors for (G) and (H). (G) LdMNPV-infected Ld652Y cells at 72 hpi. Scale bars: 20 μm. (H) Relative expression of *ie1* and *polh* in plasmid-transfected LdMNPV-infected Ld652Y cells. (∗*q* < 0.05, paired-samples *t*-test followed by Benjamini-Hochberg correction). (B and F) Red line indicates point mutations in the coiled-coil domain. (D and H) Expression level in cells transfected with the control vector (pIZ-GFP) is set as 100. Data are represented as mean with 95% confidence intervals (*n* = 3).

The functions of *bv/odv-e26* homologs were investigated using AcMNPV *Ac16* and LdMNPV *Ld127*, which are close (96.1% identity) and distant (17.3% identity) relatives of *Bm8*, respectively. GFP-fused wild-type or mutant proteins were transiently expressed in susceptible cell lines for each virus (Figures 6A and 6E). In mutant proteins, two amino acid residues corresponding to isoleucine 96 and leucine 99 of the Bm8 protein were replaced with alanine (Figures 6B and 6F). Wild-type Ac16 and Ld127 showed punctate localization in the nucleus of virus-infected cells (Figures 6C and 6G). Like the Bm8 protein, the mutant Ac16 protein showed no fluorescence, but the mutant Ld127 protein maintained GFP fluorescence after virus infection. In AcMNPV, wild-type Ac16 suppressed *ie1* and *polh* expression compared with control (*p* = 0.018 and 0.039, respectively; Figure 6D), whereas the mutant protein almost lost this suppressive activity (*p* = 0.23 and 0.19, respectively; Figure 6D). LdMNPV Ld127 significantly suppressed *ie1* and *polh* expression. Unlike group I homologs, mutant Ld127 exhibited suppressive activity, which was significantly weaker than that of wild type (Figure 6H). These results indicated that although the proteins encoded by group I and II alphabaculoviruses possess different biochemical properties, *bv/odv-e26* orthologs are conserved in most alphabaculoviruses with their suppressive activity against virus infection.

To investigate potential differences between Bm8 and Ld127 functions, we predicted their protein structures using AlphaFold^30^ (Figure S5). Both proteins harbored a long alpha-helix and a region consisting of alpha-helices and beta-sheets at the C-terminus. As some coiled-coil domain-containing proteins formed homodimer,^31^^,^^32^ we also predicted the structures of their homodimers. In these predictions, the conserved residues (i.e., isoleucine 96 and leucine 99 of Bm8 and valine 86 and leucine 89 of Ld127) are located at interface of two chains. Whereas the C-terminal regions of Bm8 did not interact with each other, those of Ld127 were closely located.

### *bv/odv-e26* is dispensable for altering locomotory pattern but required to enhance locomotory activity in BmNPV-infected *B. mori* larvae

**Figure 7:**
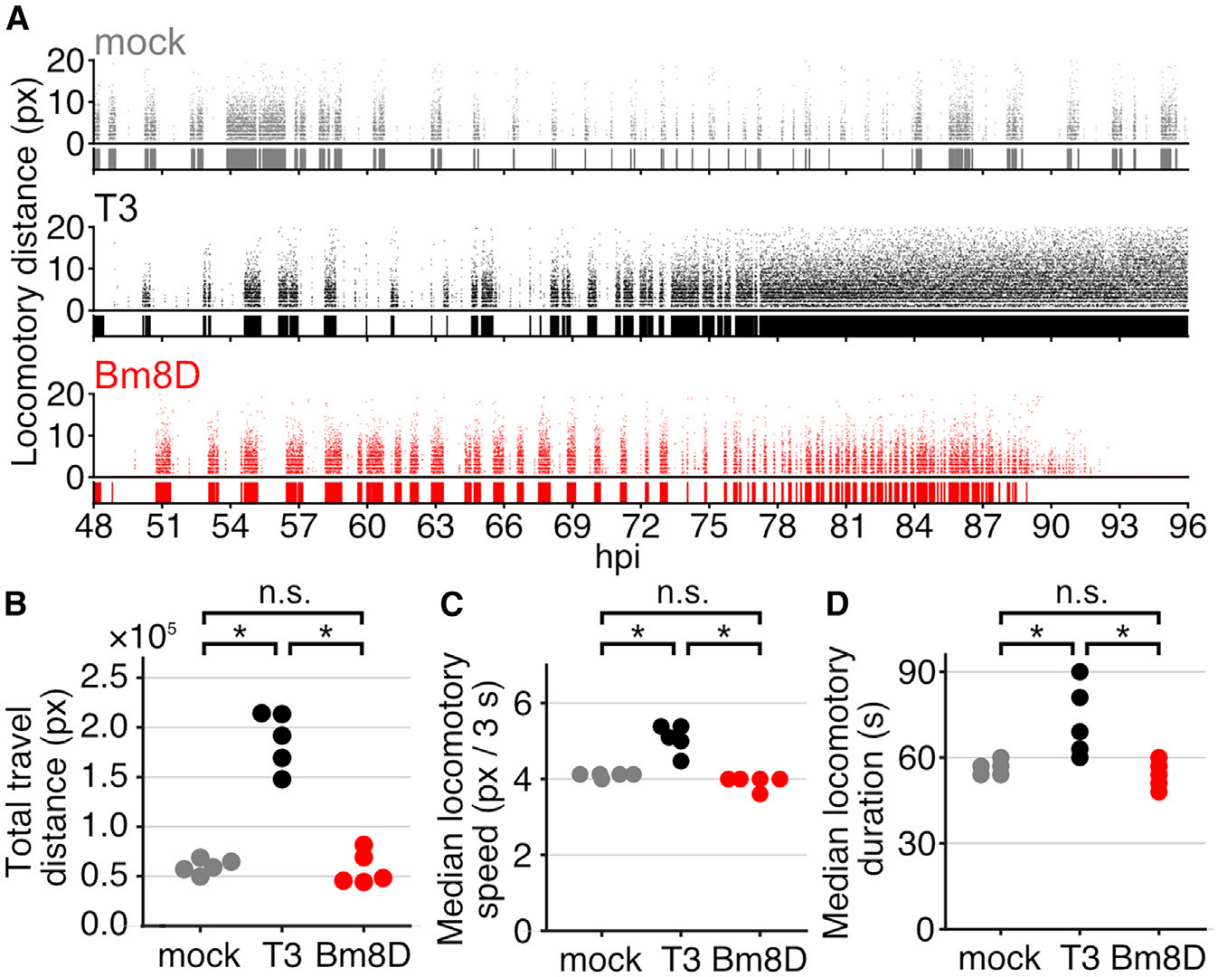
Locomotory analysis of infected larvae Locomotory activity of mock-infected (gray), T3-infected (black), and Bm8D-infected (red) larvae. (A) Temporal pattern of locomotory activity of infected larvae. The X axis shows hpi, and Y axis shows locomotory distance at 3 s interval. The diagram below each scatterplot indicates the duration of larval locomotion. Results of other individuals are shown in Figure S6. (B) Total travel distance of infected larvae. (C) Median locomotory speed of infected larvae. (D) Median locomotory duration of infected larvae. (B–D) Each point indicates an individual larva (*n* = 5) (∗*p* < 0.05, Wilcoxon rank-sum test followed by Bonferroni’s correction. n.s., no significant difference).

A previous study showed that *bv/odv-e26* is associated with virus-induced larval abnormal behavior.^25^ We examined the relationship between gene function and virus-induced host behavioral manipulation. Locomotory patterns of *B. mori* larvae infected with T3 and Bm8D were compared with those of mock-infected larvae using a high-resolution analysis system.^33^ As described previously,^34^^,^^35^ mock-infected larvae periodically repeated stational and active behaviors, whereas T3-infected larvae lost the periodical behaviors and showed continuous locomotion at a late stage of infection (Figures 7A and S6). The periodical pattern was also lost in larvae infected with Bm8D, and locomotion of Bm8D-infected larvae was continuously induced at 80–90 hpi (Figures 7A and S6). These results indicated that the locomotory pattern is altered in Bm8D-infected larvae. In contrast, the locomotion of Bm8D-infected larvae was frequently intermitted, and their locomotory activity was less enhanced, compared with those of T3-infected larvae. Consistently, Bm8D-infected larvae showed a lower travel distance than T3-infected larvae, which was almost at the same level as that of mock-infected larvae (Figure 7B). Median locomotory speed and duration of Bm8D-infected larvae were comparable with those of mock-infected larvae and significantly lower than those of T3-infected larvae (Figures 7C and 7D). These results demonstrated that loss of *Bm8* fails to enhance host locomotory activity at a late stage of infection.

**Figure 8:**
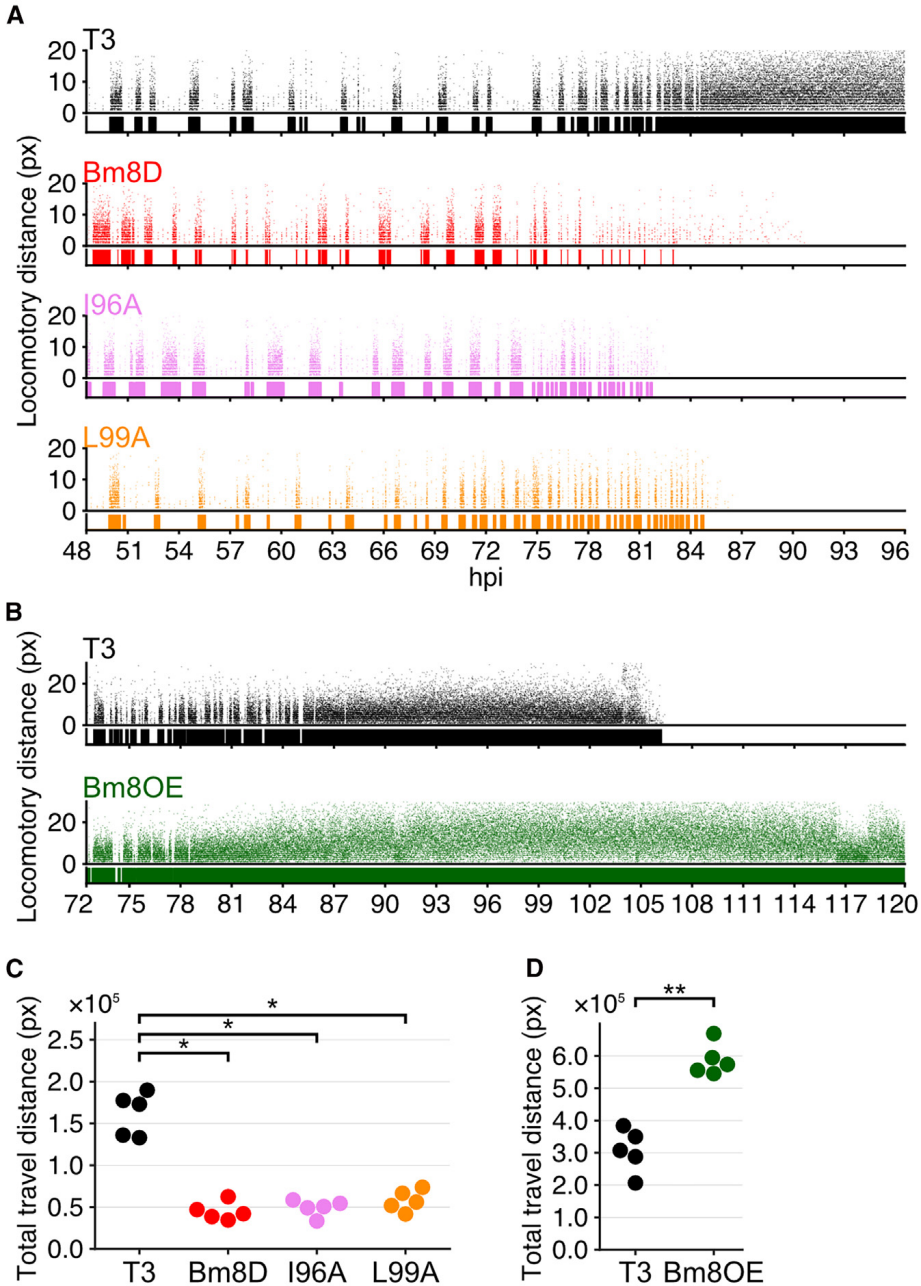
Locomotory analysis of larvae infected with *Bm8* mutants (A) Locomotory activity of T3-infected (black), Bm8D-infected (red), I96A-infected (pink), and L99A-infected (orange) larvae. (B) Locomotory activity of T3-infected (black) and Bm8OE-infected (green) larvae. (A and B) Temporal pattern of locomotory activity of infected larvae. The X axis shows hpi, and the Y axis shows locomotory distance at 3 s interval. The diagram below each scatterplot indicates the duration of larval locomotion. Results of other individuals are shown in Figure S7. (C) Total travel distance of infected larvae for viruses with point mutations in Bm8. (D) Total travel distance of Bm8OE-infected larvae. (C and D) Each point indicates an individual larva (*n* = 5) (∗*p* < 0.05, ∗∗<0.01, Wilcoxon rank-sum test followed by Bonferroni’s correction).

We further investigated the locomotory patterns of the other Bm8 mutants used in this study. Larvae infected with the recombinant viruses with point mutations in the Bm8 protein (i.e., I96A and L99A) showed similar locomotory patterns to Bm8D-infected larvae and reduced locomotory speed (Figures 8A, 8C, S7A, and S8A). Although median locomotory duration was similar between T3-and *Bm8* mutants-infected larvae (Figure S8C), T3-infected larvae exhibited long lasting locomotion at a late stage of infection which was not observed in *Bm8* mutants-infected larvae (Figures 8A and S7A). These results confirmed that loss of the Bm8 function results in the altered locomotory phenotype. As expected from reduced virulence of Bm8OE (Figures 1 and 2), Bm8OE-infected larvae showed extended abnormal locomotion (Figures 8B and S7B). Bm8OE-infected larvae also exhibited higher locomotory speed, suggesting that locomotory ability of Bm8OE-infected larvae was less affected by viral virulence factors (e.g., V-CATH) than T3-infected larvae (Figure S8B). Moreover, the initiation timing of abnormal behavior and locomotory duration were similar between T3-and Bm8OE-infected larvae (Figures 8B and S7B). As a result, Bm8OE-infected larvae exhibited longer locomotory distance than T3-infected larvae (Figure 8D). Collectively, these results suggest that inhibition of viral virulence by Bm8 has little effect on the initiation of abnormal behavior but extends the period in which larvae can move, thereby increasing locomotory distance at the late stage of infection.

## Discussion

Alphabaculoviruses exhibit systemic infection in host lepidopteran larvae, which contributes to efficient viral spreading. The present study focused on a baculoviral gene *bv/odv-e26*. This gene was shown to play crucial roles in successful systemic infection, but its function was largely unknown. Using a *Bm8*-overexpressing virus, we showed that *Bm8* overexpression delays virus infection in *B. mori* cultured cells (Figure 1). Consequently, *Bm8* overexpression prolonged host survival time accompanied by decreased activity of viral cathepsin (Figure 2). The phenotypes of the *Bm8*-overexpressing virus were opposite to those of the *Bm8*-deleted virus (Figure 2). These results demonstrate that *Bm8* suppresses viral infection in infected cells, which reduced viral virulence in host lepidopteran larvae. Knockout and overexpression of *bv/odv-e26* disturbed transcriptional regulation of BmNPV (Figure 1D). Two proteins homologous to BV/ODV-E26 examined in this study exhibited nuclear localization (Figures 4 and 6). Combined with the interaction of BV/ODV-E26 and IE1 reported in AcMNPV and BmNPV,^19^^,^^21^ BV/ODV-E26 homologs may play roles in transcriptional regulation by mediating IE1 function.

Previously, the homologs of *bv/odv-e26* were considered specific to group I alphabaculoviruses.^5^^,^^18^ In this study, however, we found that the homologous sequences of *bv/odv-e26* are widely distributed in alphabaculovirus genomes (Figure 5A). The homologs were found downstream of the *egt* locus, and the encoded proteins harbor conserved coiled-coil domains (Figure 5B). Particularly, essential residues for the suppressive function of the Bm8 protein were well conserved in group II alphabaculoviruses (Figures 3, 4, and 5B). AcMNPV Ac16 and LdMNPV Ld127, which are phylogenetically close and distant homologs of the Bm8 protein, respectively, suppressed viral gene expression in susceptible cell lines (Figure 6). These results indicated that *bv/odv-e26* homologs in a wide range of alphabaculoviruses are orthologs with conserved functions. Among alphabaculovirus genomes examined in this study, only OpbuNPV does not encode a homolog of *bv/odv-e26* (Figure 5A). Because OpbuNPV is one of the most ancestral types of alphabaculoviruses,^29^ it is suggested that *bv/odv-e26* was acquired at an early time in the course of alphabaculovirus evolution.

At 48 hpi, transiently expressed GFP-fused Bm8 with point mutations in the coiled-coil domain showed a faint GFP fluorescence, whereas GFP-fused wild-type Bm8 was visible (Figure 4). Previous study showed that the Bm8 protein level peaks at ∼24 hpi and reduces rapidly at the late stage of infection.^25^ These results suggested that Bm8 is actively degraded, and the coiled-coil domain mediates protein stability. This temporal change in Bm8 accumulation may contribute to the precise regulation of viral virulence through stage-dependent suppression. A similar dynamics was observed in the AcMNPV Ac16 protein, whereas the mutant LdMNPV Ld127 protein was stable and retained fluorescence in the nuclei as observed in wild-type Ld127 (Figure 6). Consistently, the mutant Ld127 protein had the suppressive activity, whereas Bm8 and Ac16 mutants did not (Figures 4C, 6D, and 6H). These results suggest that BV/ODV-E26 possesses different biochemical properties between group I and II viruses, which may be related to the divergence of protein sequences between them.

Coiled-coil domains consist of seven consecutive amino acid residues where 1^st^ and 4^th^ residues are hydrophobic.^36^ Isoleucine 96 and leucine 99 in Bm8 and their corresponding residues in Ac16 and Ld127 meet this criterion. Therefore, these residues may play crucial roles in protein-protein interactions. Furthermore, AlphaFold structural prediction showed that Bm8 possesses the potential to form a homodimer, where functionally conserved residues are located at the interface of the interaction (Figure S5). Structural prediction of Ld127 indicates that Ld127 can also form a homodimer but may have additional interacting regions. Although further biochemical analyses are required, Bm8 homologs might function as homodimers and two residues in the coiled-coil domain are important for their formation. The potential interacting regions at the C-terminus of Ld127 might reduce the essentiality of the two residues for interaction, resulting in different phenotypes from Bm8 and Ac16 (Figure 6).

Previous studies indicated host survival time and baculovirus-induced host behavioral manipulation are tightly correlated in multiple host-virus combinations.^37^^,^^38^^,^^39^^,^^40^ For example, fast-killing mutants of BmNPV, including *Bm8*-deleted mutants, result in short survival times, which may kill the host larvae before hijacking host behavior.^25^^,^^37^^,^^40^ This study showed that loss of Bm8 function has little effect on the induction of host abnormal behavior (Figures 7, 8, S6, and S7). However, the deletion virus failed to fully evoke enhanced locomotory activity in infected larvae compared with a wild-type virus (Figures 7, 8, and S8). These results indicate that *Bm8* is dispensable for manipulating host behavior but required for enhanced locomotory activity in infected larvae. Additionally, overexpression of *Bm8* extends abnormal behavior and may keep the larval locomotory activity higher (Figure 8). Combined with functional analysis of *bv/odv-e26* in this study, the suppressive function of *bv/odv-e26* for viral virulence may be crucial to extend host survival time for successful host behavioral manipulation. We also demonstrated that *bv/odv-e26* is widely conserved in alphabaculoviruses. Further studies of this gene in other host-virus combinations may advance the understanding of molecular mechanisms underlying baculovirus-induced host behavioral manipulation.

### Limitations of the study

This study revealed that BV/ODV-E26 has little effect on the induction of abnormal behavior. Thus, the exact triggers of the host’s abnormal behavior remain to be elusive. Also, detailed mechanisms of action of BV/ODV-E26, including actual protein-protein interactions during the viral infection, have to be analyzed in further studies.

## Resource availability

### Lead contact

Requests for further information and resources should be directed to and will be fulfilled by the lead contact, Hiroyuki Hikida (hhikida@scl.kyoto-u.ac.jp).

### Materials availability

All unique/stable reagents generated in this study are available from the lead contact.

### Data and code availability

#### Data

The RNA sequencing data are available at DDBJ. The analyzed data are available at Kyoto University Research Information Repository. Accession numbers are listed in the key resources table.

#### Code

This paper dose not report original code.

#### Additional information

Any additional information required to reanalyze the data reported in this paper is available from the lead contact upon request.

## Acknowledgments

We thank Noriko Matsuda-Imai, Takashi Kiuchi, and Toru Shimada for helpful comments and Munetaka Kawamoto for clerical assistance. LdMNPV A21 and AcMNPV C6 strain were kindly provided from Motoko Ikeda at Nagoya University and Masashi Iwanaga at Utsunomiya University, respectively. Ld652Y cells were kindly provided from Motoko Ikeda and Masataka G. Suzuki at the University of Tokyo. BmNPV Bm8 antibody was kindly provided from WonKyung Kang at RIKEN. Molecular graphics and analyses performed with UCSF ChimeraX, developed by the Resource for Biocomputing, Visualization, and Informatics at the University of California, San Francisco, with support from 10.13039/100000002National Institutes of Health R01-GM129325 and the Office of Cyber Infrastructure and Computational Biology, 10.13039/100000060National Institute of Allergy and Infectious Diseases. This study was supported by Japan Society for the Promotion of Science (10.13039/501100000646JSPS) KAKENHI grant number 16H05051 and 19H02966 to S.K., 19J13438 to H.H., and 24H02290 to S.K. and H.H. H.H. was a JSPS Research Fellow (DC2).

## Author contributions

H.H. and S.K. conceptualized the study and wrote drafts of the manuscript. H.H. and R.K. performed the experiments. H.H., R.K., and S.K. analyzed the data.

## Declaration of interests

The authors declare no conflict of interests.

## STAR★Methods

### Key resources table

**Table:** 

REAGENT or RESOURCE	SOURCE	IDENTIFIER
**Antibodies**		

BmNPV V-CATH antibody	Daimon et al.^26^	
BmNPV Bm8 antibody	Imai et al.^20^	

**Bacterial and virus strains**		

BmNPV T3	Maeda et al.^41^	
AcMNPV C6	Possee et al.^42^	
LdMNPV A21	Slavicek et al.^43^	
BmNPV-abb	Zhou et al.^44^	
BmNPV Bm8D	Imai et al.^20^	
BmNPV Bm8OE	This study	
BmNPV Bm8-L88A	This study	
BmNPV Bm8-I96A	This study	
BmNPV Bm8-L99A	This study	

**Chemicals, peptides, and recombinant proteins**		

E−64	Cayman	

**Critical commercial assays**		

Pierce BCA Protein Assay Kit	Thermo Fisher Scientific	

**Deposited data**		

RNA sequencing data	DDBJ	PRJDB13017
Locomotory data	Kyoto University Research Information Repository	https://doi.org/10.57723/274185

**Experimental models: Cell lines**		

*Bombyx mori:* BmN-4	Maintained in our laboratory	
*Spodoptera frugiperda:* Sf9	Maintained in our laboratory	
*Lymantria dispar:* Ld652Y	Prof. Motoko Ikeda	

**Experimental models: Organisms/strains**		

Bombyx mori: Kinshu × Showa	Ueda sanshu	

**Oligonucleotides**		

See Table S1 for primers		

**Recombinant DNA**		

pIZ-GFP	Kawaoka et al.^45^	
pBh-EPS	Kang et al.^46^	
Bm8region-pcDNA	Hikida et al.^24^	
pIZ-V5/His-Bm8LG	This study	
pIZ-V5/His-Bm8LGcc	This study	
pIZ-V5/His-Ac16LG	This study	
pIZ-V5/His-Ac16LGcc	This study	
pIZ-V5/His-Ld127LG	This study	
pIZ-V5/His-Ld127LGcc	This study	

**Software and algorithms**		

BLAST+ (v2.10.0)	Camacho et al.^47^	
Hisat2 (v2.1.0)	Kim et al.^48^	
SAMtools (v1.9)	Li et al.^49^	
BEDTools (v2.29.0)	Quinlan et al.^50^	
DESeq2 (v1.26.0)	Love et al.^51^	
MAFFT (v7.453)	Katoh et al.^52^	
IQ-TREE (v1.6.12)	Minh et al.^53^	
Needle	Madeira et al.^54^	
AlphaFold server	Abramson et al.^30^	alphafoldserver.com
UCSF ChimeraX (v1.8)	Meng et al.^55^	

### Experimental model and study participant details

#### Insects, cultured cells, and viruses

*B. mori* larvae (Kinshu × Showa) were reared at 25°C with an artificial diet. BmN-4, Sf9, and Ld652Y cells were maintained with TC 100 medium supplemented with 10% fetal bovine serum. BmNPV T3,^41^ AcMNPV C6,^42^ and LdMNPV A21^43^ strains were used as wild-type viruses. Bm8D^20^ was used as the *Bm8*-deleted BmNPV. BmNPV titers were determined using the plaque assay, and AcMNPV and LdMNPV titers were determined using the 50% tissue culture infectious dose (TCID_50_) method.

### Method details

#### Generation of recombinant viruses

Bm8OE, a *Bm8*-overexpressing virus, was generated as follows. The genomic region containing the *Bm8* coding sequence and its promoter was PCR-amplified from the genome of BmNPV T3 (Genbank: accession no. L33180.1). A nucleotide sequence coding a DYKDDDDK tag was attached at the 3′ terminal of the *Bm8* coding sequence. The SV40 polyA sequence was PCR-amplified from the plasmid pIZ-GFP.^45^ These two amplified fragments were inserted into the pBh-EPS vector, which carries the *polh* gene and its flanking regions,^46^^,^^56^ at the PstI site using the In-Fusion HD Cloning Kit (Takara). The resultant plasmid and Bsu36I-digested BmNPV-abb^44^ genome DNA were cotransfected into BmN-4 cells using FuGENE HD (Promega). The recombinant virus was purified two times using the plaque assay by identifying OB-producing plaques. *Bm8* overexpression was validated by quantitative polymerase chain reaction (qPCR) and western blotting (Figure S9).

Functional domains in Bm8 were searched using InterPro 102.0 (www.ebi.ac.uk/interpro/, accessed October 5, 2024).^57^ Bm8 homologs were searched using BLASTP version 2.10.0 against the National Center for Biotechnology Information (NCBI) nr database,^47^ and their coiled-coil domains were aligned with MAFFT version 7.453.^52^ Point mutations of the conserved amino acid residues in the coiled-coil domain were introduced into the plasmid Bm8region-pcDNA^24^ using KOD -Plus- Mutagenesis Kit (Toyobo). The resultant plasmids were cotransfected with Bsu36I-digested Bm8D genome DNA, and recombinant viruses were purified as described above by identifying white plaques.

All primers used are listed in Table S1.

#### Viral virulence in infected larvae

Newly molted fifth-instar *B. mori* larvae were starved for several hours, injected intrahemocoelically with a viral suspension containing 10^5^ plaque-forming units (PFUs), and returned to an artificial diet at 25°C (*n* = 20). Dead and surviving larvae were counted from 96 hpi until all larvae died at 6 h intervals. Median lethal time (LT_50_) was determined using the Kaplan–Meier estimate.

#### Western blotting

Newly molted fifth-instar *B. mori* larvae were injected with a viral suspension as described above. Hemolymph and fat bodies were collected at 90 hpi from three larvae and mixed into a single tube. Hemolymph was diluted with 2× sample buffer (100 mM Tris-HCl [pH 6.8], 4% sodium dodecyl sulfate, 12% 2-mercaptoethanol, 20% glycerol, and bromophenol blue). Fat bodies were ground in RIPA buffer (50 mM Tris-HCl [pH 8.0], 100 mM NaCl, 3 mM MgCl_2_, 1% NP-40, and cOmplete Protease Inhibitor Cocktail (Roche)) with 30 μg/mL E64 (Cayman Chemical), a cysteine protease inhibitor, followed by centrifugation to remove the insoluble fraction. The protein concentration of the supernatant was measured by Pierce BCA Protein Assay Kit (Thermo Fisher Scientific). Total protein was adjusted and diluted with 2× sample buffer. Following the previous study,^58^ a portion of the protein samples was subjected to western blotting using a BmNPV V-CATH antibody.^26^

To confirm Bm8 overexpression, BmN-4 cells were infected with BmNPV at a multiplicity of infection (MOI) of 5 and harvested at 24 hpi. The cells were resuspended with TBN buffer (10 mM Tris-HCl [pH 6.8], 140 mM NaCl, 3 mM MgCl_2_, 0.5% NP-40, and cOmplete Protease Inhibitor Cocktail (Roche)), and insoluble fraction was collected, followed by resuspension by RIPA buffer. The collected fraction was added to the same amount of 2× sample buffer, sheared by a 27 G syringe 10 times, and subjected to western blotting using a BmNPV Bm8 antibody.^20^

#### Cathepsin assay

V-CATH activity was measured following previous studies (*n* = 3).^58^^,^^59^^,^^60^ The hemolymph of infected larvae was collected at the 90 hpi and centrifuged to remove cells. The supernatant was added to 125 mM phosphate buffer [pH 7.0] with 1.1% azocasein and incubated for 3 h at 37°C in the presence or absence of E-64 (Cayman Chemical). After incubation, 10% trichloroacetic acid was added to the solution, followed by centrifuging at 12,000 rpm for 5 min at room temperature to remove proteins. The enzymatic activity was measured as absorbance at 405 nm.

#### Quantification of viral genomic DNA

BmN-4 cells were infected with BmNPV at an MOI of 5 and harvested at 0, 6, 12, 24, 48, and 72 hpi. Total DNA was extracted, and the amount of viral DNA was measured as described previously.^61^

#### Quantification of viral gene expression

Total RNA was prepared from BmNPV-infected BmN-4 cells with TRI reagent (Cosmo Bio Co., Ltd.), according to the manufacturer’s protocol. First-strand cDNA was synthesized from 1 μg total RNA using SuperScript IV (Thermo Fisher Scientific), and qPCR was performed using the KAPA SYBR FAST qPCR Kit (Kapa Biosystems) with the primers listed in Table S1. Expression was calculated using the 2^−ΔCt^ method and represented as relative expression when the expression in T3-infected cells at 72 hpi was 100.

#### RNA sequencing

BmNPV-infected BmN-4 cells were harvested at 48 hpi, and total RNA was extracted as described above. Library preparation and sequencing were performed by Novagen Co., Ltd (China) using Novaseq 6000 with the 150 bp paired-end mode. The quality of sequence data was examined with FastQC. The reads were mapped to the BmNPV genome (GenBank accession no. L33180.1) using Hisat2 version 2.1.0.^48^ The resultant SAM files were converted to BAM files using SAMtools version 1.9 and subsequently converted to BED files using BEDTools version 2.29.0.^49^^,^^50^ Viral gene expression was normalized to reads per kilobase of transcript per million, using mapped viral reads as the total number of reads.

Sequencing was performed using three biological replicates, but the third datasets were clustered by replicate rather than treatment (Figure S1A), which suggests that differential expression analysis would be affected by this replicate. Because the other two replicates were clustered by virus treatment (Figure S1B), differential expression analysis was performed using the first and second replicates using DESeq2 version 1.26.0,^51^ and the gene expression pattern was independently verified by the third replicate (Figure S1C).

#### Microscopic observation

Newly molted fifth-instar *B. mori* larvae were injected with a viral suspension as described above. MSGs were dissected from infected larvae at 90 hpi and washed in phosphate-buffered saline. The dissected tissues were observed under a stereomicroscope (Zeiss Axio Zoom.V16, Carl Zeiss).

#### Transient expression of *bv/odv-e26*

Genes homologous to *bv/odv-e26* were PCR-amplified from the genomes of BmNPV T3, AcMNPV C6, and LdMNPV A21. The *gfp* coding region was PCR-amplified from the plasmid pIZ-GFP. These fragments were inserted into the pIZ-V5/His vector (Invitrogen) using In-Fusion HD Cloning Kit (Takara). Mutations in the coiled-coil domain were introduced by PCR with the primers listed in Table S1. The resultant plasmids were transfected into cultured cells susceptible to the original viruses using X-tremeGENE HP DNA Transfection Reagent (Roche). Transfection and infection experiments were performed with different parameters depending on virus–host cell combinations. For BmNPV, cells were infected with the viruses at an MOI of 5 PFU at 48 h post transfection (hpt). For AcMNPV and LdMNPV, cells were infected at an MOI of 5 TCID_50_ at 24 hpt. Expression levels of *ie1* and *polh* were measured and *bv/odv-e26* overexpression was validated by real-time qPCR as described above. Cells were observed at the designated time points (Figures 3C, 4C, 6C, and 6G) under a fluorescence microscope (FLoid Cell Imaging Station, Thermo Fischer Scientific).

#### Homology search

ORFs located between *egt* and *Bm9* homologs were selected from the genomes of alphabaculoviruses registered in the NCBI GenBank database. Their homologous genes were searched against alphabaculovirus genomes using BLASTP.^47^ Homologous relationships were defined by the reciprocal best hit. Network analysis was further performed. First, a graph was constructed, where genes and homologous relationships comprised nodes and edges, respectively (Figure S3). In this graph, genes connected to *bv/odv-e26* in BmNPV were considered as *bv/odv-e26* homologs. All protein sequences of *bv/odv-e26* homologs were aligned with MAFFT version 7.453.^52^ Phylogenetic trees were constructed using IQ-TREE^53^ multicore version 1.6.12 under the LG model and the JTT+F+I + G4 model for concatenated core gene products and BV/ODV-E26, respectively, with 1000 ultrafast bootstraps. The constructed trees were visualized using iTOL version 5.^62^ The dataset of concatenated sequences of baculovirus core genes was retrieved from Harrison et al.^2^ Identity between Bm8 and Ac16 or Ld127 was calculated using the Needle program on EMBOSS web service.^54^ Protein structures of Bm8 and Ld127 were predicted using AlphaFold server (alphafoldserver.com)^30^ and visualized using UCSF ChimeraX version 1.8.^55^

#### Locomotion assay

Locomotion assays were performed as reported previously.^34^ Newly molted fourth-instar larvae were injected with a viral suspension or control medium as described above. At designated time points, each larva (*n* = 5) was placed in a 100 mm cell culture dish with a portion of an artificial diet. Locomotion was recorded at 3 s intervals using a time-lapse camera (TLC200-pro, Brinno) under continuous light conditions for 48 h. The video was processed, and larval locomotory patterns and activities were analyzed using KaicoTracker, a software modified from a previous method.^33^

### Quantification and statistical analysis

#### Statistical analysis

Host survival time was compared using the pairwise log rank test. V-CATH activity between treatments with DMSO and E64 was compared by one-sided paired-samples *t*-test. Viral genome replication and temporal gene expression were compared using Tukey’s HSD test. In the transient expression experiment, viral gene expression was compared using the paired-samples *t*-test, and *p*-values were corrected to *q*-values using the Benjamini–Hochberg method. Locomotory distance, speed, and duration were compared using the Wilcoxon rank-sum test, followed by Bonferroni’s correction. The statistical details are shown in the figure legends.

## References

[1] Rohrmann G.F. (2019). https://www.ncbi.nlm.nih.gov/books/NBK543458/.

[2] Harrison R.L., Herniou E.A., Jehle J.A., Theilmann D.A., Burand J.P., Becnel J.J., Krell P.J., van Oers M.M., Mowery J.D., Bauchan G.R., Ictv Report Consortium (2018). ICTV virus taxonomy profile: Baculoviridae. J. Gen. Virol..

[3] Jehle J.A., Blissard G.W., Bonning B.C., Cory J.S., Herniou E.A., Rohrmann G.F., Theilmann D.A., Thiem S.M., Vlak J.M. (2006). On the classification and nomenclature of baculoviruses: A proposal for revision. Arch. Virol..

[4] Wennmann J.T., Keilwagen J., Jehle J.A. (2018). Baculovirus Kimura two-parameter species demarcation criterion is confirmed by the distances of 38 core gene nucleotide sequences. J. Gen. Virol..

[5] Herniou E.A., Luque T., Chen X., Vlak J.M., Winstanley D., Cory J.S., O’Reilly D.R. (2001). Use of whole genome sequence data to infer baculovirus phylogeny. J. Virol..

[6] Blissard G.W., Theilmann D.A. (2018). Baculovirus entry and egress from insect cells. Annu. Rev. Virol..

[7] Keddie B.A., Aponte G.W., Volkman L.E. (1989). The pathway of infection of *Autographa californica* nuclear polyhedrosis virus in an insect host. Science.

[8] Engelhard E.K., Kam-Morgan L.N., Washburn J.O., Volkman L.E. (1994). The insect tracheal system: a conduit for the systemic spread of *Autographa californica* M nuclear polyhedrosis virus. Proc. Natl. Acad. Sci. USA.

[9] Barrett J.W., Brownwright A.J., Primavera M.J., Palli S.R. (1998). Studies of the nucleopolyhedrovirus infection process in insects by using the green fluorescence protein as a reporter. J. Virol..

[10] Ohkawa T., Majima K., Maeda S. (1994). A cysteine protease encoded by the baculovirus *Bombyx mori* nuclear polyhedrosis virus. J. Virol..

[11] Hawtin R.E., Zarkowska T., Arnold K., Thomas C.J., Gooday G.W., King L.A., Kuzio J.A., Possee R.D. (1997). Liquefaction of *Autographa californica* nucleopolyhedrovirus-infected insects is dependent on the integrity of virus-encoded chitinase and cathepsin genes. Virology.

[12] Guarino L.A., Summers M.D. (1986). Interspersed homologous DNA of *Autographa californica* nuclear polyhedrosis virus enhances delayed-early gene expression. J. Virol..

[13] Guarino L.A., Summers M.D. (1988). Functional mapping of *Autographa california* nuclear polyhedrosis virus genes required for late gene expression. J. Virol..

[14] Guarino L.A., Xu B., Jin J., Dong W. (1998). A virus-encoded RNA polymerase purified from baculovirus-infected cells. J. Virol..

[15] Fuchs L.Y., Woods M.S., Weaver R.F. (1983). Viral transcription during Autographa californica nuclear polyhedrosis virus infection: a novel RNA polymerase induced in infected *Spodoptera frugiperda* Cells. J. Virol..

[16] McLachlin J.R., Miller L.K. (1994). Identification and characterization of *vlf-1*, a baculovirus gene involved in very late gene expression. J. Virol..

[17] Graves L.P., Hughes L.C., Irons S.L., Possee R.D., King L.A. (2019). In cultured cells the baculovirus P10 protein forms two independent intracellular structures that play separate roles in occlusion body maturation and their release by nuclear disintegration. PLoS Pathog..

[18] Kang W. (2009). Molecular dissection of *Bombyx mori* nucleopolyhedrovirus *orf8* gene. Virol. Sin..

[19] Nie Y., Fang M., Theilmann D.A. (2009). AcMNPV AC16 (DA26, BV/ODV-E26) regulates the levels of IE0 and IE1 and binds to both proteins via a domain located within the acidic transcriptional activation domain. Virology.

[20] Imai N., Kurihara M., Matsumoto S., Kang W.K. (2004). *Bombyx mori* nucleopolyhedrovirus *orf8* encodes a nucleic acid binding protein that colocalizes with IE1 during infection. Arch. Virol..

[21] Kang W., Imai N., Kawasaki Y., Nagamine T., Matsumoto S. (2005). IE1 and *hr* facilitate the localization of *Bombyx mori* nucleopolyhedrovirus ORF8 to specific nuclear sites. J. Gen. Virol..

[22] Beniya H., Braunagel S.C., Summers M.D. (1998). *Autographa californica* Nuclear Polyhedrosis Virus: Subcellular Localization and Protein Trafficking of BV/ODV-E26 to Intranuclear Membranes and Viral Envelopes. Virology.

[23] Kang W., Katsuma S., Matsuda-Imai N., Kurihara M., Yoshiga T., Shimada T., Matsumoto S. (2012). Identification and Characterization of Host Factors Interacting with *Bombyx mori* Nucleopolyhedrovirus ORF8. J. Microbiol..

[24] Hikida H., Kokusho R., Kobayashi J., Shimada T., Katsuma S. (2018). Inhibitory role of the Bm8 protein in the propagation of Bombyx mori nucleopolyhedrovirus. Virus Res..

[25] Katsuma S., Kobayashi J., Koyano Y., Matsuda-Imai N., Kang W., Shimada T. (2012). Baculovirus-encoded protein BV/ODV-E26 determines tissue tropism and virulence in lepidopteran insects. J. Virol..

[26] Daimon T., Katsuma S., Shimada T. (2007). Mutational analysis of active site residues of chitinase from *Bombyx mori* nucleopolyhedrovirus. Virus Res..

[27] Hoover K., Grove M., Gardner M., Hughes D.P., McNeil J., Slavicek J. (2011). A Gene for an Extended Phenotype. Science.

[28] Nie Y., Theilmann D.a. (2010). Deletion of AcMNPV AC16 and AC17 results in delayed viral gene expression in budded virus infected cells but not transfected cells. Virology.

[29] Harrison R.L., Rowley D.L., Mowery J.D., Bauchan G.R., Burand J.P. (2017). The *Operophtera brumata* nucleopolyhedrovirus (OpbuNPV) represents an early, divergent lineage within genus *Alphabaculovirus*. Viruses.

[30] Abramson J., Adler J., Dunger J., Evans R., Green T., Pritzel A., Ronneberger O., Willmore L., Ballard A.J., Bambrick J. (2024). Accurate structure prediction of biomolecular interactions with AlphaFold 3. Nature.

[31] Joslyn G., Richardson D.S., White R., Alber T. (1993). Dimer formation by an N-terminal coiled coil in the APC protein. Proc. Natl. Acad. Sci. USA.

[32] Nikolay R., Wiederkehr T., Rist W., Kramer G., Mayer M.P., Bukau B. (2004). Dimerization of the human E3 ligase CHIP via a coiled-coil domain is essential for its activity. J. Biol. Chem..

[33] Hikida H., Katsuma S. (2022). KaicoTracker: a robust and automated locomotory analysis for baculovirus-infected silkworm larvae. J. Insect Biotechnol. Sericol..

[34] Hikida H., Katsuma S. (2021). igh-resolution analysis of baculovirus-induced host manipulation in the domestic silkworm, Bombyx mori. Parasitology.

[35] Kômoto N. (2017). Behavior of the larvae of wild mulberry silkworm *Bombyx mandarina*, domesticated silkworm *B. mori* and their hybrid. J. Insect Biotechnol. Sericol..

[36] Mason J.M., Arndt K.M. (2004). Coiled coil domains: stability, specificity, and biological implications. Chembiochem.

[37] Katsuma S., Shimada T. (2015). The killing speed of *egt*-inactivated *Bombyx mori* nucleopolyhedrovirus depends on the developmental stage of *B. mori* larvae. J. Invertebr. Pathol..

[38] Han Y., van Houte S., Drees G.F., van Oers M.M., Ros V.I.D. (2015). Parasitic manipulation of host behaviour: Baculovirus SeMNPV EGT facilitates tree-top disease in *Spodoptera exigua* larvae by extending the time to death. Insects.

[39] Ros V.I.D., van Houte S., Hemerik L., van Oers M.M. (2015). Baculovirus-induced tree-top disease: How extended is the role of *egt* as a gene for the extended phenotype?. Mol. Ecol..

[40] Hikida H., Kokusho R., Matsuda-Imai N., Katsuma S. (2020). Bombyx mori nucleopolyhedrovirus Bm96 suppresses viral virulence in *Bombyx mori* larvae. J. Invertebr. Pathol..

[41] Maeda S., Kawai T., Obinata M., Fujiwara H., Horiuchi T., Saeki Y., Sato Y., Furusawa M. (1985). Production of human alpha-interferon in silkworm using a baculovirus vector. Nature.

[42] Possee R.D., Sun T.P., Howard S.C., Ayres M.D., Hill-Perkins M., Gearing K.L. (1991). Nucleotide sequence of the *Autographa californica* nuclear polyhedrosis 9.4 kbp EcoRI-I and -R (Polyhedrin gene) region. Virology.

[43] Slavicek J.M., Podgwaite J., Lanner-Herrera C. (1992). Properties of two *Lymantria dispar* nuclear polyhedrosis virus isolates obtained from the microbial pesticide Gypchek. J. Invertebr. Pathol..

[44] Zhou C.E., Ko R., Maeda S. (1998). Polyhedron-like inclusion body formation by a mutant nucleopolyhedrovirus expressing the granulin gene from a granulovirus. Virology.

[45] Kawaoka S., Mitsutake H., Kiuchi T., Kobayashi M., Yoshikawa M., Suzuki Y., Sugano S., Shimada T., Kobayashi J., Tomari Y., Katsuma S. (2012). A role for transcription from a piRNA cluster in de novo piRNA production. RNA.

[46] Kang W., Tristem M., Maeda S., Crook N.E., O’Reilly D.R. (1998). Identification and characterization of the Cydia pomonella granulovirus cathepsin and chitinase genes. J. Gen. Virol..

[47] Camacho C., Coulouris G., Avagyan V., Ma N., Papadopoulos J., Bealer K., Madden T.L. (2009). BLAST+: architecture and applications. BMC Bioinf..

[48] Kim D., Paggi J.M., Park C., Bennett C., Salzberg S.L. (2019). Graph-based genome alignment and genotyping with HISAT2 and HISAT-genotype. Nat. Biotechnol..

[49] Li H., Handsaker B., Wysoker A., Fennell T., Ruan J., Homer N., Marth G., Abecasis G., Durbin R., 1000 Genome Project Data Processing Subgroup (2009). The Sequence Alignment/Map format and SAMtools. Bioinformatics.

[50] Quinlan A.R., Hall I.M. (2010). BEDTools: a flexible suite of utilities for comparing genomic features. Bioinformatics.

[51] Love M.I., Huber W., Anders S. (2014). Moderated estimation of fold change and dispersion for RNA-seq data with DESeq2. Genome Biol..

[52] Katoh K., Standley D.M. (2013). MAFFT multiple sequence alignment software version 7: improvements in performance and usability. Mol. Biol. Evol..

[53] Minh B.Q., Schmidt H.A., Chernomor O., Schrempf D., Woodhams M.D., von Haeseler A., Lanfear R. (2020). IQ-TREE 2: New models and efficient methods for phylogenetic inference in the genomic era. Mol. Biol. Evol..

[54] Madeira F., Pearce M., Tivey A.R.N., Basutkar P., Lee J., Edbali O., Madhusoodanan N., Kolesnikov A., Lopez R. (2022). Search and sequence analysis tools services from EMBL-EBI in 2022. Nucleic Acids Res..

[55] Meng E.C., Goddard T.D., Pettersen E.F., Couch G.S., Pearson Z.J., Morris J.H., Ferrin T.E. (2023). UCSF ChimeraX: Tools for structure building and analysis. Protein Sci..

[56] Kokusho R., Kawamoto M., Koyano Y., Sugano S., Suzuki Y., Shimada T., Katsuma S. (2015). *Bombyx mori* nucleopolyhedrovirus actin rearrangement-inducing factor 1 enhances systemic infection in *B. mori* larvae. J. Gen. Virol..

[57] Paysan-Lafosse T., Blum M., Chuguransky S., Grego T., Pinto B.L., Salazar G.A., Bileschi M.L., Bork P., Bridge A., Colwell L. (2023). InterPro in 2022. Nucleic Acids Res..

[58] Nakanishi T., Goto C., Kobayashi M., Kang W., Suzuki T., Dohmae N., Matsumoto S., Shimada T., Katsuma S. (2010). Comparative studies of lepidopteran baculovirus-specific protein FP25K: Development of a novel *Bombyx mori* Nucleopolyhedrovirus-based vector with a modified *fp25K* gene. J. Virol..

[59] Katsuma S., Tanaka S., Shimada T., Kobayashi M. (2004). Reduced cysteine protease activity of the hemolymph of *Bombyx mori* larvae infected with *fp25K*-inactivated *Bombyx mori* nucleopolyhedrovirus results in the reduced postmortem host degradation. Arch. Virol..

[60] Slack J.M., Kuzio J., Faulkner P. (1995). Characterization of *v-cath*, a cathepsin L-like proteinase expressed by the baculovirus *Autographa californica* multiple nuclear polyhedrosis virus. J. Gen. Virol..

[61] Katsuma S., Kokusho R. (2017). A conserved glycine residue is required for proper functioning of a baculovirus VP39 protein. J. Virol..

[62] Letunic I., Bork P. (2021). Interactive Tree Of Life (iTOL) v5: an online tool for phylogenetic tree display and annotation. Nucleic Acids Res..

