# Supplementary material for: BV/ODV-E26 is a conserved baculoviral inhibitory factor for optimizing viral virulence in lepidopteran hosts
Source: iScience. 2024 Dec 31;28(2):111723. doi: 10.1016/j.isci.2024.111723 (PMC11787618; doi:10.1016/j.isci.2024.111723)
Supplement: Document S1. Figures S1–S9 and Tables S1 and S2 and Data S1 and S2 [file mmc1.pdf]

iScience, Volume *28*

Supplemental information

BV/ODV-E26 is a conserved baculoviral inhibitory factor for optimizing viral virulence in lepidopteran hosts

Hiroyuki Hikida, Ryuhei Kokusho, and Susumu Katsuma

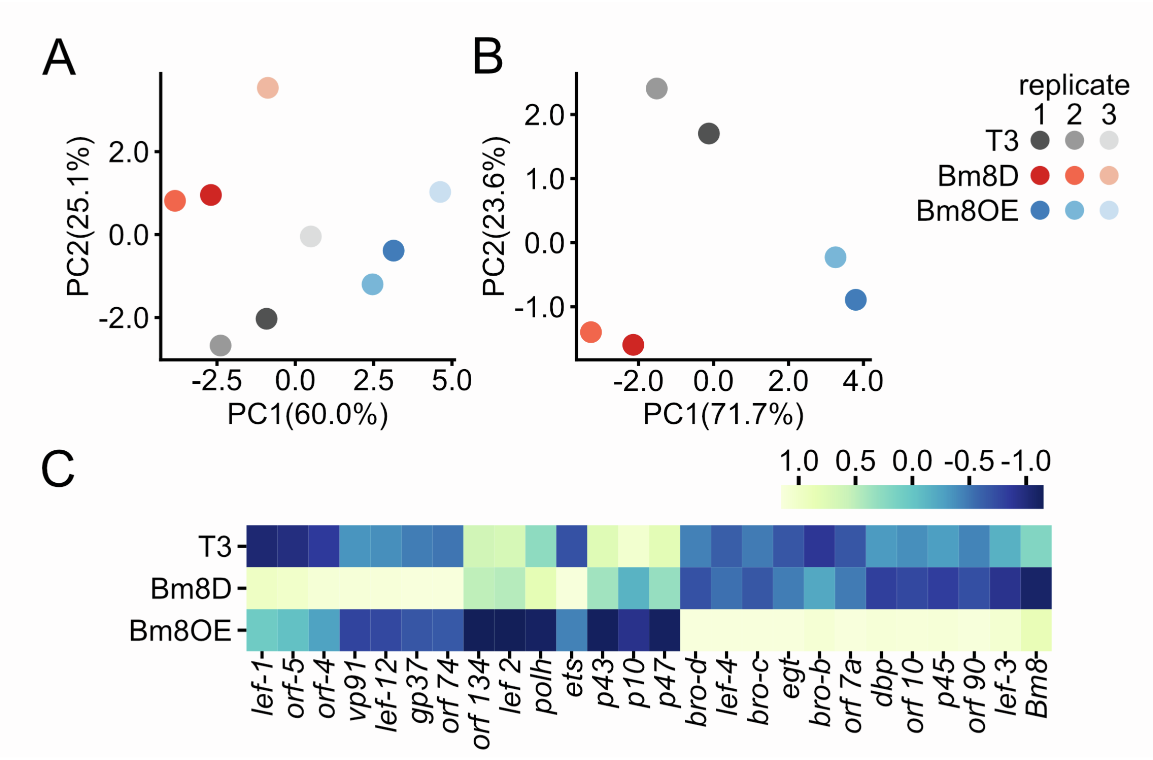

Fig S1. RNA-seq analysis.

(A) Principal component analysis (PCA) results using three replicates. (B) PCA results using replicates 1 and 2. (A and B) Colored circles indicate individual replicates. The percentage shown on each axis indicates the contribution of each principal component. (C) Heat map of viral gene expression in replicate 3. The order of genes corresponds to those in Fig. 1D.

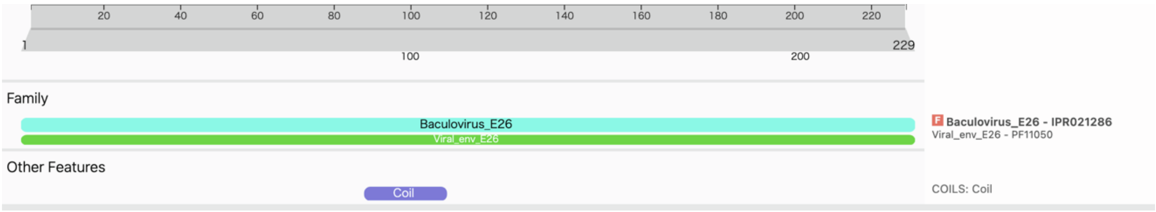

Fig S2. Prediction of functional domains in the Bm8 protein.

An output image from InterPro 102.0. Entire sequences are assigned to the protein family consisting of BV/ODV-E26 homologs. A coiled-coil domain was predicted from 89th to 109th amino acid residues.

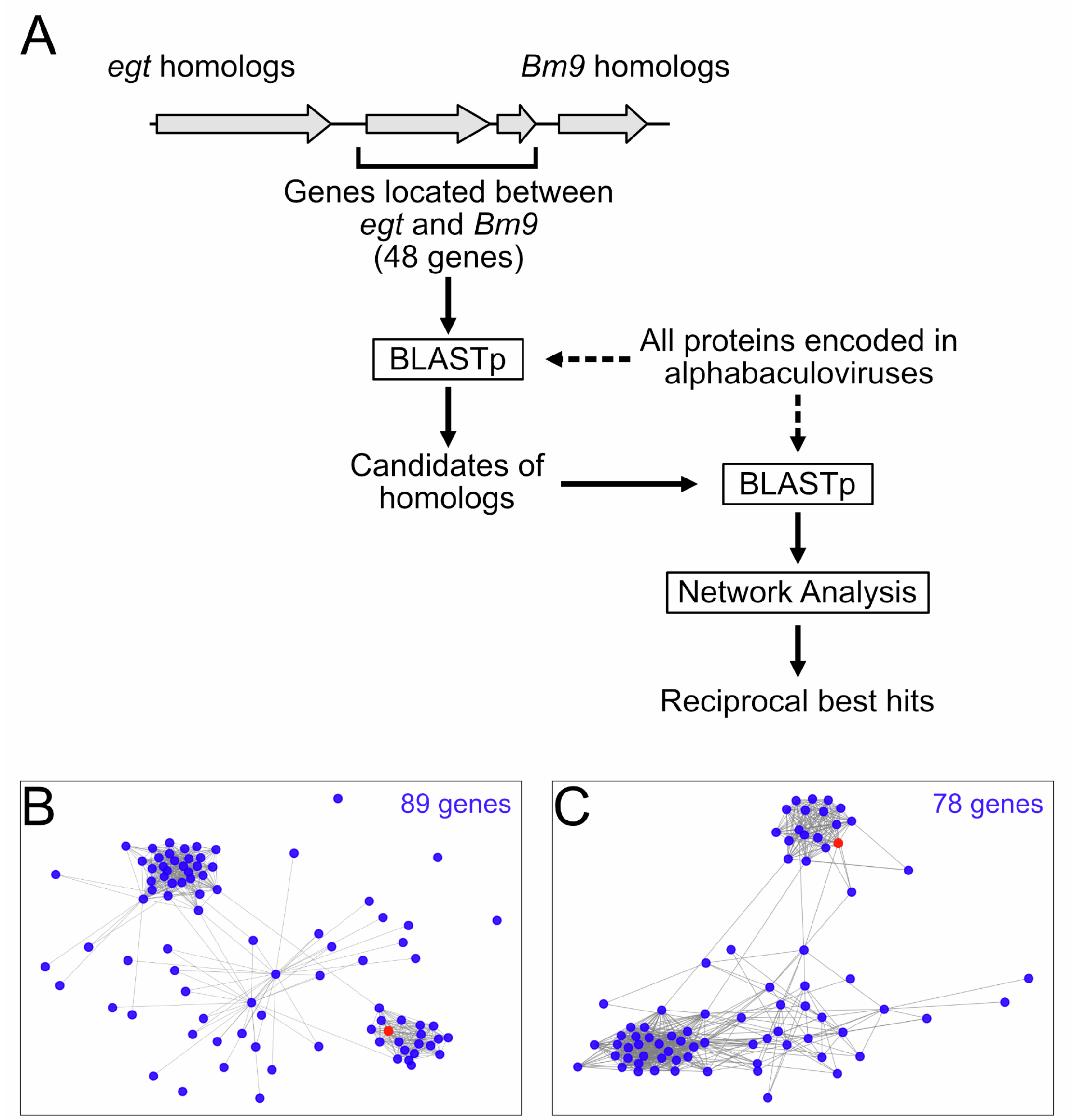

Fig S3. Identification of *bv/odv-e26* homologs.

(A) Scheme of gene-order-based identification of *bv/odv-e26* homologs. (B) Network of homology relationship in 89 candidate homologs. (C) Network of the same relationship in 78 candidates showing the reciprocal best hits. (B and C) Each node and edge indicate gene and homology relationship, respectively. The red node represents *Bm8*.

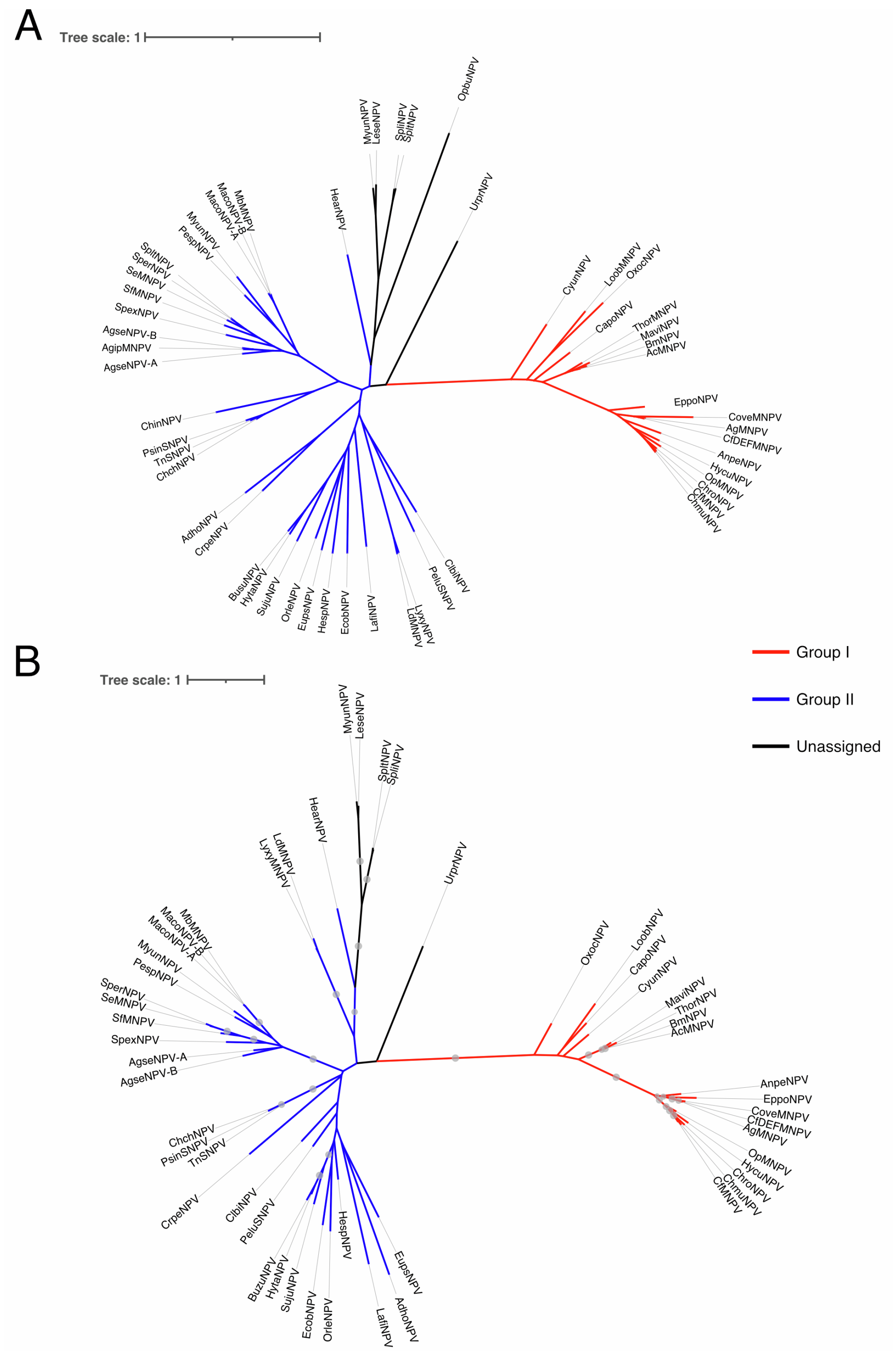

Fig S4. Phylogenetic analysis of *bv/odv-e26* homologs.

Unrooted phylogenic trees of (A) alphabaculoviruses, constructed based on 38 core genes with the LG substitution model and (B) *bv/odv-e26* homologs, constructed under the JTT+F+I+G4 model with 1000 ultrafast bootstraps. Red, blue, and black indicate group I, group II, and unassigned alphabaculoviruses, respectively. Gray circles indicate bootstrap values > 90. The abbreviations of virus names are listed in Table S2.

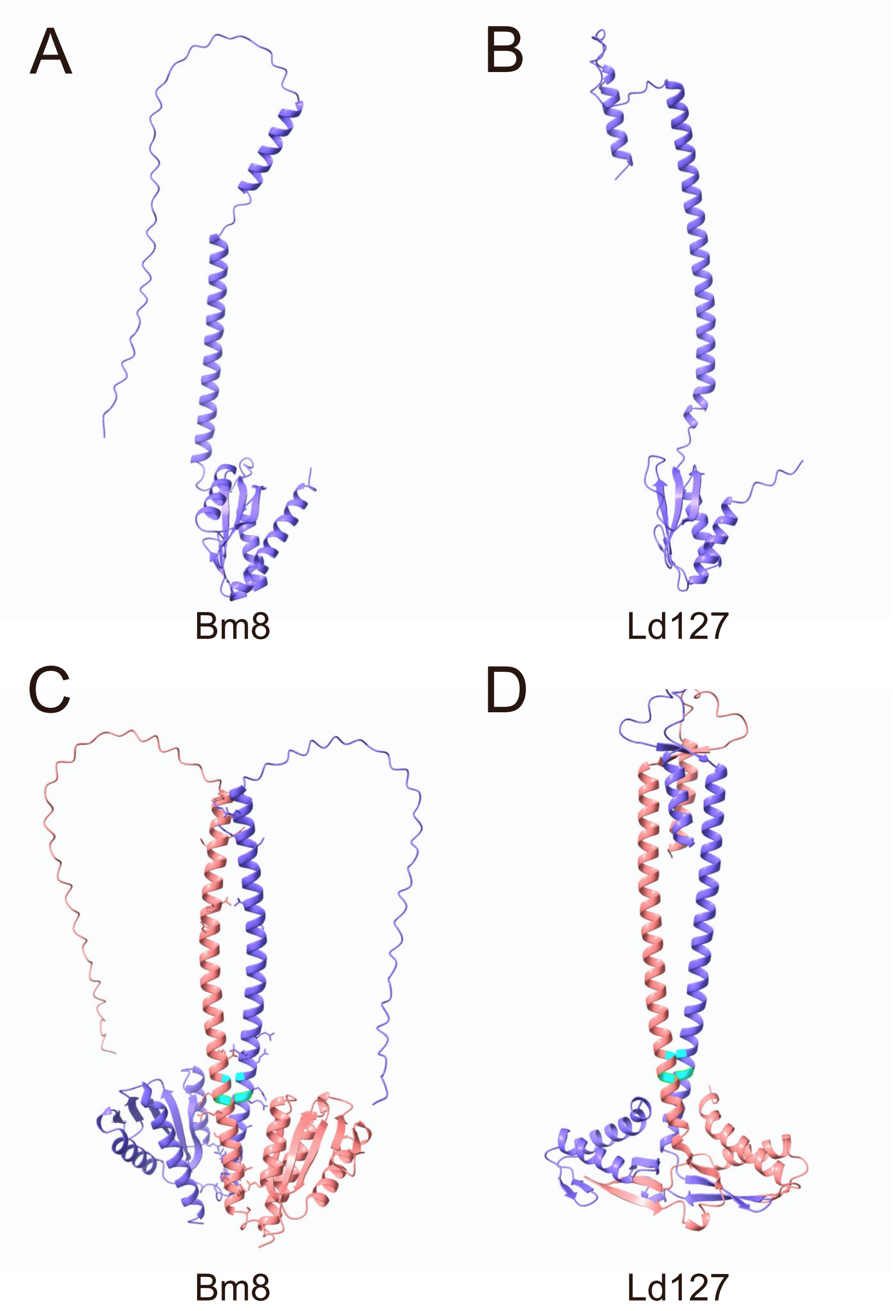

Fig S5. Predicted structures of Bm8 and Ld127.

Bm8 and Ld127 structures predicted by AlphaFold. Monomers of (A) Bm8 and (B) Ld127. Homodimers of (C) Bm8 and (D) Ld127. (C, D) Each color represents each chain. (C) Isoleucine 96 and leucine 99 are highlighted by cyan. (D) Valine 86 and leucine 89 are highlighted by cyan.

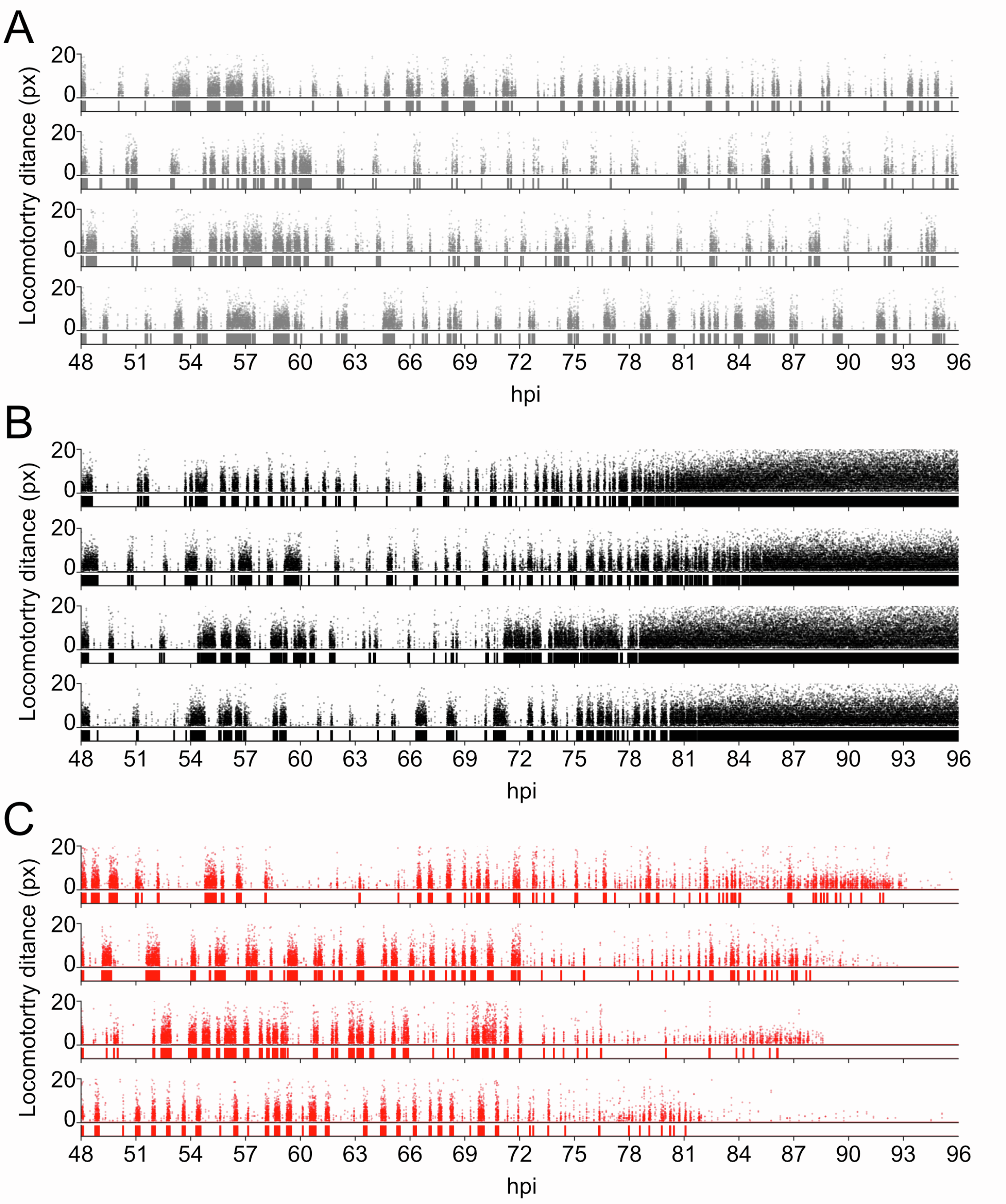

Fig S6. Behavioral patterns of larval individuals not shown in Fig. 7.
(A) Mock-infected, (B) T3-infected, and (C) Bm8D-infected larvae.

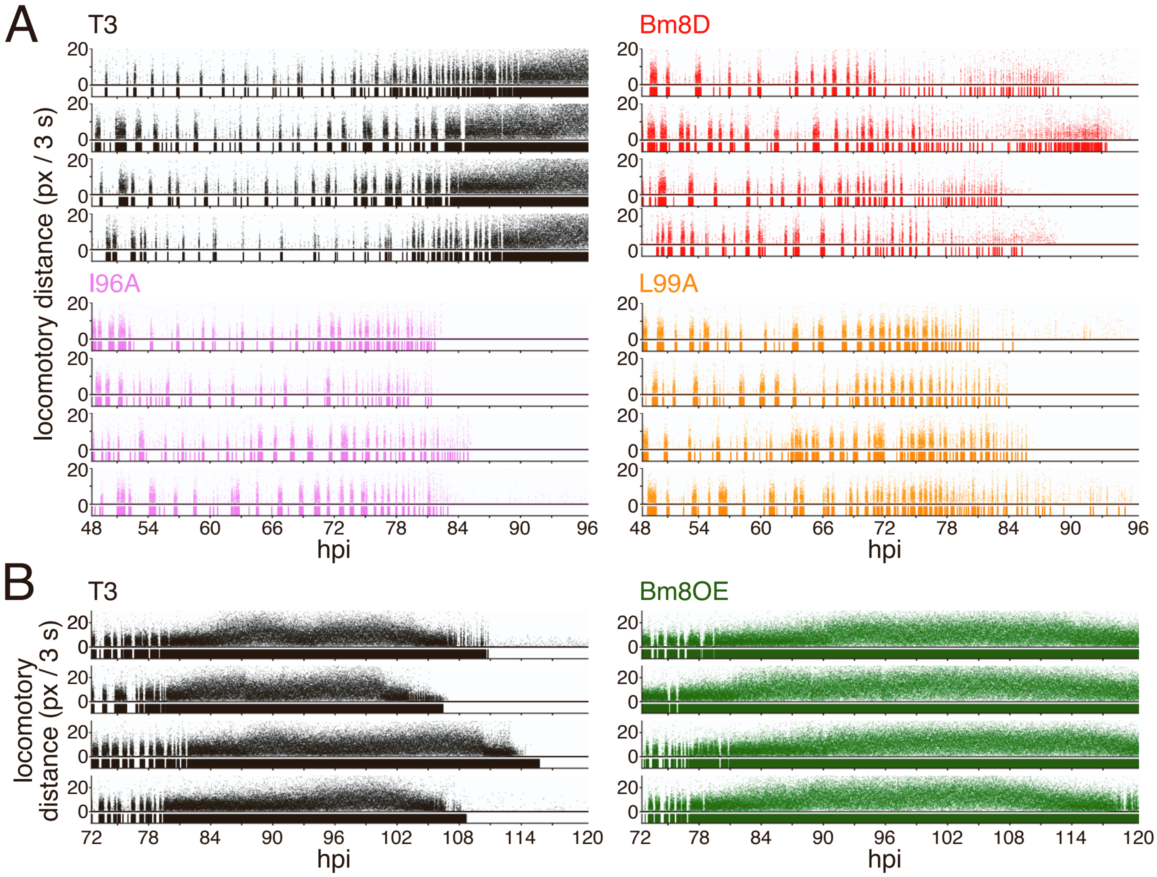

Fig S7. Behavioral patterns of larval individuals not shown in Fig. 8.

(A) Larvae infected with T3, Bm8D and viruses with point mutations in Bm8. Locomotion was observed from 48 to 96 hpi. (B) Larvae infected with T3 and Bm8OE. Locomotion was observed from 72 to 120 hpi.

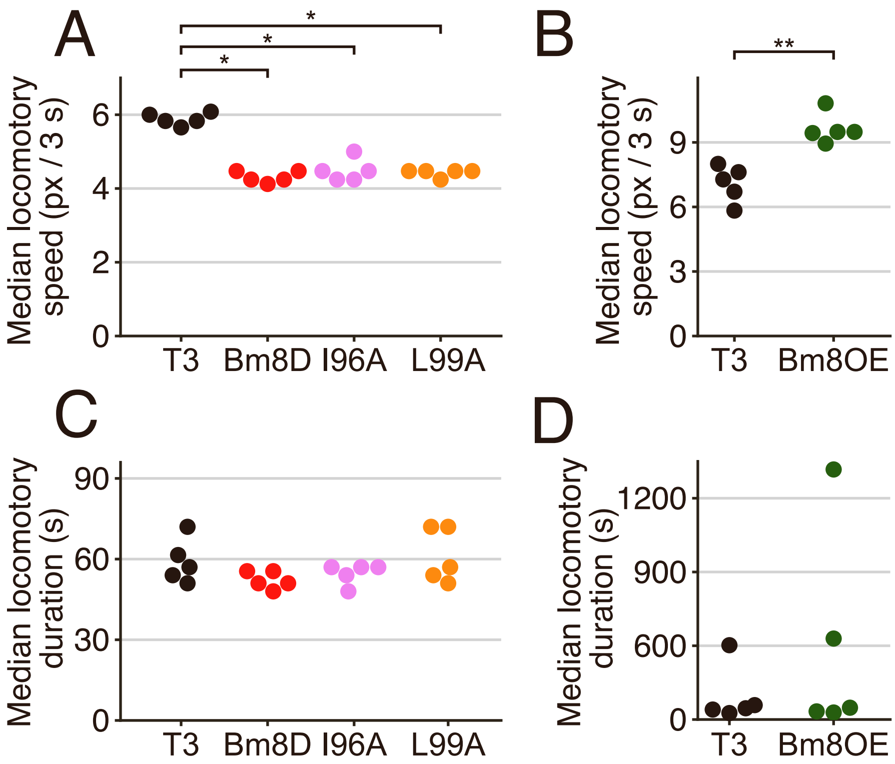

Fig S8. Locomotory analysis of *Bm8* mutants.

Median locomotory speed of the larvae infected with (A) T3, Bm8D, and viruses with point mutations in Bm8 and (B) T3 and Bm8OE. Median locomotory duration of the larvae infected with (C) T3, Bm8D, and viruses with point mutations in Bm8 and (D) T3 and Bm8OE. (A–D) Each point indicates an individual larva (*$p < 0.05$, **<0.01. Wilcoxon rank sum test followed by Bonferroni's correction).

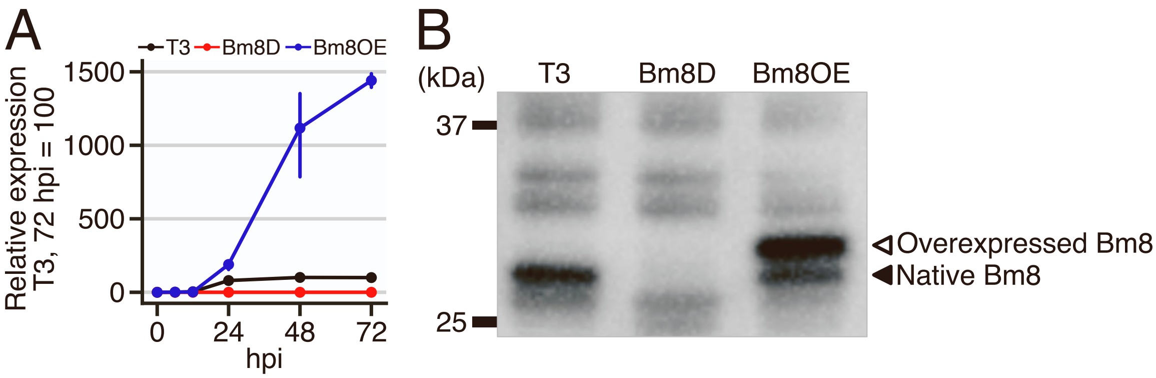

Fig S9. Confirmation of Bm8 overexpression.

(A) Temporal expression of *Bm8*. Data are represented as mean with 95% confidence intervals (n = 3). (B) Expression of the Bm8 protein in cultured cells at 24 hpi. Black and empty arrowheads indicate the size of native Bm8 and overexpressed Bm8 with a DYKDDDDK tag, respectively. The uncropped image and Coomassie Brilliant Blue (CBB) staining of the loading gel were shown in Supplementary Data.

Table S1. Primer list.

Name	Use	Nucleotide Sequene (5′→3′)
Bm8-FLAG-F-iP	Generation of Bm8OE	GACGATGACAAGTAATTTGAAGGGTGAGGAAGAGCCCAATTG
Bm8-FLAG-R-iP	Generation of Bm8OE	ATCCTTGTAGTCATAGGCGTTAATATCACTTTGAGATTCATCTTGC
Bm8FLAG-F-IF	Generation of Bm8OE	ATTTGTATCGGAGCTCGATTTACGTAAGTTTTGGG
Bm8FLAG-R-IF	Generation of Bm8OE	CTGCAATAAACAAGTTTACTTGTCATCGTCATCC
sv40pA-F-IF	Generation of Bm8OE	ACTTGTTTATTGCAGCTTATAATG
sv40pA-R-IF	Generation of Bm8OE	ATAAATGTCAGAATTACAGACATGATAAGATACATTGATGAG
Bm8regionF	Sequence confrimation	CTCGAGGAACCTACATCCCATATTTGACAAC
Bm8regionR	Sequence confrimation	AAGCTTGTACAATTCGTGTGTCAATGATCTC
Bm8L88A-F	Generation of Bm8-L88A	CTAAGGAAAAAGAACGAAATTATTGC
Bm8L88A-R	Generation of Bm8-L88A	CGCATTTTGCAAATGCAGCAGTTTC
Bm8I96A/L99A-F	Generation of Bm8-I96A and Bm8-L99A	GTTAGAAAACTCGAAAGTGCAC
Bm8I96A-R	Generation of Bm8-I96A	CAACTCGGCAGCAATTTCGTTCTTTTTCC
Bm8L99A-R	Generation of Bm8-L99A	CGCCTCGGCAATAATTTCGTTCTTTTTCC
Bm8F	Construction of pIZ-Bm8LG	GTACCGAGCTCGGATCCATGAATTCTGTTCACACGCG
Bm8-gfpR	Construction of pIZ-Bm8LG	ACTGCCTCCACCGCCAGAGCCACCTCCACCATAGGCGTTAATATCACTTTG
gfpF	Construction of pIZ-Bm8LG	GGCGGTGGAGGCAGTGGAGGTGGCGGATCGTTGGTGAGCAAGGGCGAGGAG
gfpR	Construction of pIZ-Bm8LG	CTGGACTAGTGGATCCTCACTTGTACAGCTCGTCCATGCC
Bm8/Ac16-gfpR	Construction of pIZ-Bm8LG, pIZ-Ac16LG	ACTGCCTCCACCGCCAGAGCCACCTCCACCATAGGCGTTAATATCACTTTG
Ac16F	Construction of pIZ-Ac16LG	GTACCGAGCTCGGATCCATGGAGTCTGTTCAAACGCG
Ld127F	Construction of pIZ-Ld127	GTACCGAGCTCGGATCCATGTCGACTTGGAGGAACAAATTGC
Ld127-gfpR	Construction of pIZ-Ld127	ACTGCCTCCACCGCCAGAGCCACCTCCACCGTGCCGCCGGCGGCG
Bm8CCmutF	Introducing mutaitons in coiled-coil domain	GAACGAAATTGCTGCCGAGGCGGTTAGAAAACTC
Bm8CCmutR	Introducing mutaitons in coiled-coil domain	GAGTTTTCTAACCGCCTCGGCAGCAATTTCGTTC
Ac16CCmutF	Introducing mutaitons in coiled-coil domain	GAAAAAAGAACGAAATTGCTGCCGAGGCGGTTAGAAAACTTG
Ac16CCmutR	Introducing mutaitons in coiled-coil domain	CAAGTTTTCTAACCGCCTCGGCAGCAATTTCGTTCTTTTTTC
Ld127CCmutF	Introducing mutaitons in coiled-coil domain	GGACCGCTCGCGCAGACCGAGCGCGCCGCGAGCAC
Ld127CCmutR	Introducing mutaitons in coiled-coil domain	GTGCTCGCGGCGCGCTCGGTCTGCGCGAGCGGTCC
ago3_gF	Quantification of host genome	TTTCTTAGTACACTCAAACG
ago3_gR	Quantification of host genome	CTCTCTTCGTAGAACATATC
polhqF	Quantification of *polh* and viral genome	GAACAAGAGGAGAAGCAATG
polhqR	Quantification of *polh* and viral genome	TCCAGTTGGCGATTAACTTC
ie1qF	Quantification of *ie1*	TACTTGGACGATTCACAAAG
ie1qR	Quantification of *ie1*	GTGCAAATGTTCGTGTTGTG
lef2qF	Quantification of *lef2*	ACATGCTGAACAGCAAGATC
lef2qR	Quantification of *lef2*	ACATCGGTTTTCACATTTGG
vp39qF	Quantification of *vp39*	ACTTTTCATGATGTCACTGC
vp39qR	Quantification of *vp39*	AGTACTTGCAAATCGACACG
Bm8qF	Quantification of *Bm8*	AACTCGAAAGTGCACAGAAG
Bm8qR	Quantification of *Bm8*	CAATAATTGTGCGAATTGTG
Ac_ie1qF	Quantification of AcMNPV *ie1*	TCACGTACAAATACAGCAGCGTCG
Ac_ie1qR	Quantification of AcMNPV *ie1*	CATGCTGCCGTCCTCCTTCTTAAC
Ac_polhqF	Quantification of AcMNPV *polh*	GTTACAAATTCCTGGCCCAACAC
Ac_polhqR	Quantification of AcMNPV *polh*	ATGCGGTACTCGTTGTTGCTG
Ac16qF	Quantification of *Ac16*	TGCACAGAAGAAGACAACGCAC
Ac16qR	Quantification of *Ac16*	CGGCCAAACGTCTCCTTACAAAC
Ld_ie1qF	Quantification of LdMNPV *ie1*	CCCGTCGAACTTGTTGATGATGTC
Ld_ie1qR	Quantification of LdMNPV *ie1*	AAGAACGAGGAGCGCCTGAC
Ld_polhqF	Quantification of LdMNPV *polh*	AAAGCACTTGGAACAGCACGAG
Ld_polhqR	Quantification of LdMNPV *polh*	GGCTTGACATTGCGGATCTCTTTG
Ld127qF	Quantification of *Ld127*	ATTGGCGCGTGATTTCGGTG
Ld127qR	Quantification of *Ld127*	ATATTGTAGGTTGCACCGCTCC

Table S2. List of alphabaculovirus genomes used in this study.

Species	Isolate	Accession number	Virus Abbrev.
Adoxophyes honmai nucleopolyhedrovirus	ADN001	AP006270	AdhoNPV
Agrotis ipsilon multiple nucleopolyhedrovirus	Illinois	EU839994	AgipMNPV
Agrotis segetum nucleopolyhedrovirus A	Polish	DQ123841	AgseNPV-A
Agrotis segetum nucleopolyhedrovirus B	English	KM102981	AgseNPV-B
Antheraea pernyi nucleopolyhedrovirus	Liaoning	DQ486030	AnpeNPV
Anticarsia gemmatalis multiple nucleopolyhedrovirus	2D	DQ813662	AgMNPV
Autographa californica multiple nucleopolyhedrovirus	C6	L22858	AcMNPV
Bombyx mori nucleopolyhedrovirus	T3	L33180	BmNPV
Buzura suppressaria nucleopolyhedrovirus	Hubei	KF611977	BuzuNPV
Catopsilia pomona nucleopolyhedrovirus	416	KU565883	CapoNPV
Choristoneura fumiferana DEF multiple nucleopolyhedrovirus		AY327402	CfDEFMNPV
Choristoneura fumiferana multiple nucleopolyhedrovirus	Ireland	AF512031	CfMNPV
Choristoneura murinana nucleopolyhedrovirus	Darmstadt	KF894742	ChmuNPV
Choristoneura rosaceana nucleopolyhedrovirus	NB_1	KC961304	ChroNPV
Chrysodeixis chalcites nucleopolyhedrovirus		AY864330	ChchNPV
Chrysodeixis includens nucleopolyhedrovirus	IE	KJ631622	PsinSNPV
Clanis bilineata nucleopolyhedrovirus	DZ1	DQ504428	ClbiNPV
Condylorrhiza vestigialis nucleopolyhedrovirus	PR.2002	KJ631623	CoveMNPV
Cryptophlebia peltastica nucleopolyhedrovirus	SA	MH394321	CrpeNPV
Cyclophragma undans nucleopolyhedrovirus	Whiov	KT957089	CyunNPV
Ectropis obliqua nucleopolyhedrovirus	A1	DQ837165	EcobNPV
Epiphyas postvittana nucleopolyhedrovirus		AY043265	EppoNPV
Euproctis pseudoconspersa nucleopolyhedrovirus	Hangzhou	FJ227128	EupsNPV
Helicoverpa armigera nucleopolyhedrovirus	G4	AF271059	HearNPV
Hemileuca species nucleopolyhedrovirus	MEM	KF158713	HespNPV
Hyphantria cunea nucleopolyhedrovirus	N9	AP009046	HycuNPV
Hyposidra talaca nucleopolyhedrovirus	India.001	MH261376	HytaNPV
Lambdina fiscellaria nucleopolyhedrovirus	GR15	KP752043	LafiNPV
Leucania separata nucleopolyhedrovirus	AH1	AY394490	LeseNPV
Lonomia obliqua nucleopolyhedrovirus	SP/2000	KP763670	LoobNPV
Lymantria dispar multiple nucleopolyhedrovirus	5-6	AF081810	LdMNPV
Lymantria xylina nucleopolyhedrovirus	5	GQ202541	LyxyMNPV
Mamestra brassicae multiple nucleopolyhedrovirus	K1	JQ798165	MbMNPV
Mamestra configurata nucleopolyhedrovirus A	90/2	U59461	MacoNPV-A
Mamestra configurata nucleopolyhedrovirus B	96B	AY126275	MacoNPV-B
Maruca vitrata nucleopolyhedrovirus	MV-8	EF125867	MaviNPV
Mythimna unipuncta nucleopolyhedrovirus A	#7	MF375894	MyunNPV
Mythimna unipuncta nucleopolyhedrovirus B	KY310	MH124167	MyunNPV
Operophtera brumata nucleopolyhedrovirus	MA	MF614691	OpbuNPV
Orgyia leucostigma nucleopolyhedrovirus	CFS-77	EU309041	OrleNPV
Orgyia pseudotsugata multiple nucleopolyhedrovirus		U75930	OpMNPV
Oxyplax ochracea nucleopolyhedrovirus	435	MF143631	OxocNPV
Peridroma saucia nucleopolyhedrovirus	GR167	KM009991	PespNPV
Perigonia lusca nucleopolyhedrovirus		KM596836	PeluSNPV
Spodoptera eridania nucleopolyhedrovirus	251	MH320559	SperNPV
Spodoptera exempta nucleopolyhedrovirus	244.1	MH717816	SpexNPV
Spodoptera exigua multiple nucleopolyhedrovirus	US1	AF169823	SeMNPV
Spodoptera frugiperda multiple nucleopolyhedrovirus	3AP2	EF035042	SfMNPV
Spodoptera littoralis nucleopolyhedrovirus	AN1956	JX454574	SpliNPV
Spodoptera litura nucleopolyhedrovirus	G2	AF325155	SpltNPV
Sucra jujuba nucleopolyhedrovirus	473	KJ676450	SujuNPV
Thysanoplusia orichalcea nucleopolyhedrovirus	p2	JX467702	ThorNPV
Trichoplusia ni single nucleopolyhedrovirus		DQ017380	TnSNPV
Urbanus proteus nucleopolyhedrovirus	Southern Brazil	KR011717	UrprNPV

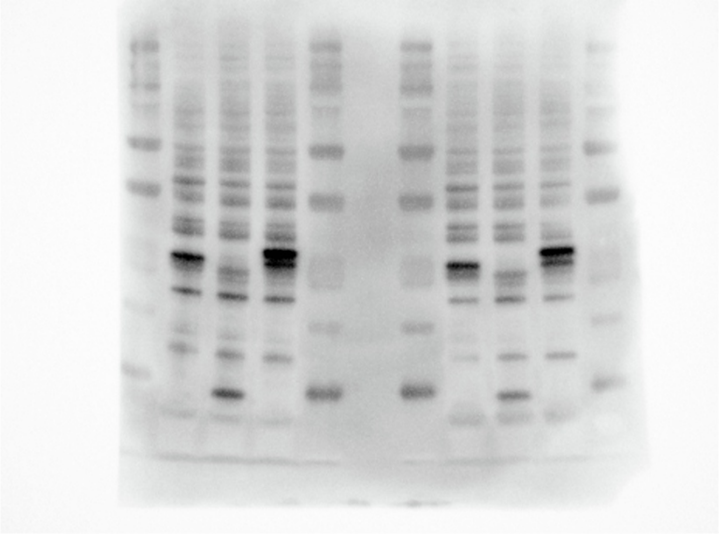

Supplementary Data 1. Full-size image of Fig. S9B.

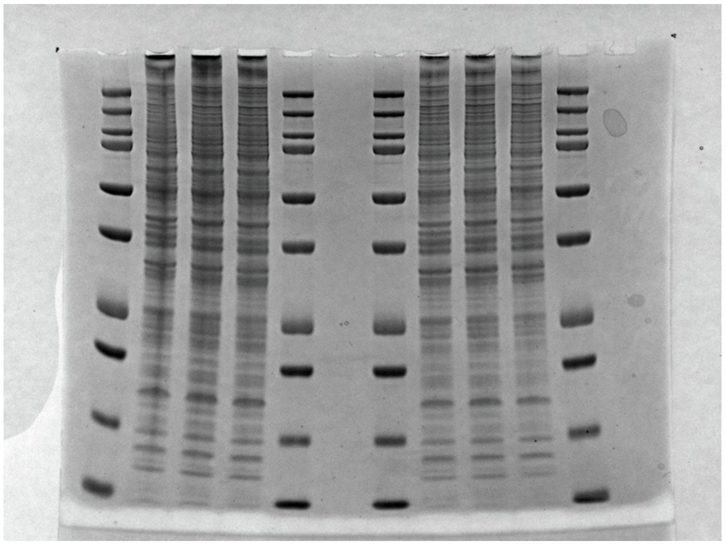

Supplementary Data 2. CBB staining of the gel used for Fig. S9B.
